# Combined transcriptomic and metabolomic analysis of alginate oligosaccharides alleviating salt stress in rice seedlings

**DOI:** 10.1186/s12870-023-04470-x

**Published:** 2023-09-29

**Authors:** You-Wei Du, Ling Liu, Nai-Jie Feng, Dian-Feng Zheng, Mei-Ling Liu, Hang Zhou, Peng Deng, Ya-xing Wang, Hui-Min Zhao

**Affiliations:** 1 College of Coastal Agriculture Sciences, Guangdong Ocean University, Zhanjiang, 524088 China; 2 South China Center of National Saline-tolerant Rice Technology Innovation Center, Zhanjiang, 524088 China; 3 Shenzhen Research Institute of Guangdong Ocean University, Shenzhen, 518108 China

**Keywords:** Rice, Salt stress, Alginate oligosaccharides, Transcriptome, Metabolome

## Abstract

**Background:**

Salt stress is one of the key factors limiting rice production. Alginate oligosaccharides (AOS) enhance plant stress resistance. However, the molecular mechanism underlying salt tolerance in rice induced by AOS remains unclear. FL478, which is a salt-tolerant indica recombinant inbred line and IR29, a salt-sensitive rice cultivar, were used to comprehensively analyze the effects of AOS sprayed on leaves in terms of transcriptomic and metabolite profiles of rice seedlings under salt stress.

**Results:**

In this experiment, exogenous application of AOS increased SOD, CAT and APX activities, as well as GSH and ASA levels to reduce the damage to leaf membrane, increased rice stem diameter, the number of root tips, aboveground and subterranean biomass, and improved rice salt tolerance. Comparative transcriptomic analyses showed that the regulation of AOS combined with salt treatment induced the differential expression of 305 and 1030 genes in FL478 and IR29. The expressed genes enriched in KEGG pathway analysis were associated with antioxidant levels, photosynthesis, cell wall synthesis, and signal transduction. The genes associated with light-trapping proteins and RLCK receptor cytoplasmic kinases, including CBA, LHCB, and Lhcp genes, were fregulated in response to salt stress. Treatment with AOS combined with salt induced the differential expression of 22 and 50 metabolites in FL478 and IR29. These metabolites were mainly related to the metabolism of amino and nucleotide sugars, tryptophan, histidine, and β -alanine. The abundance of metabolites associated with antioxidant activity, such as 6-hydroxymelatonin, wedelolactone and L-histidine increased significantly. Combined transcriptomic and metabolomic analyses revealed that dehydroascorbic acid in the glutathione and ascorbic acid cycles plays a vital role in salt tolerance mediated by AOS.

**Conclusion:**

AOS activate signal transduction, regulate photosynthesis, cell wall formation, and multiple antioxidant pathways in response to salt stress. This study provides a molecular basis for the alleviation of salt stress-induced damage by AOS in rice.

**Supplementary Information:**

The online version contains supplementary material available at 10.1186/s12870-023-04470-x.

## Background

Rice (*Oryza sativa* L.) is an essential food crop and the staple food of more than half of the world’s population [1, 2]. Soil salinization has become a major challenge to global agricultural production, affecting more than 20% of the world’s farmland [3]. Salt stress usually causes osmotic stress, ion stress induced by excessive accumulation of Na+, and oxidative damage due to high levels of reactive oxygen species (ROS) [4, 5]. Salt stress often elicits plant stress response [6]. Stress induces signal transduction and production of ROS-scavenging enzymes and non-enzymatic antioxidants, and osmotic regulators to mitigate salt stress damage [5, 7]. For example, the SOS pathway is activated under salt stress to facilitate the reverse transport of Na + ions from the cytoplasm to the plasmodesmata [8]. Different levels of salt stress increase CAT, POD, APX, GST in rice [9]. Salt stress significantly increased the levels of soluble protein, proline, and soluble sugar in cotton leaves [10].

Alginate oligosaccharides (AOS) are degradation products of sodium alginate [11] that contribute to plant stress resistance [12] by scavenging hydroxyl radicals and superoxide anions [13], reducing the damage of superoxide dismutase (SOD) and peroxidase (POD) under salt stress to enhance the stress resistance [14]. AOS also regulates plant hormones. For example, AOS relieves osmotic stress by regulating LEA1, psbA, SnRK2 and P5CS via ABA signaling pathway [15].

Recent transcriptomic and metabolomic analyses have identified metabolic pathways and their regulatory genes [16]. For example, high temperatures can upregulate genes specifically involved in the metabolism of rate-limiting enzymes, shikimic acid, lignin and MVA in the glycolysis of rice flag leaves, resulting in the accumulation of specific secondary metabolites [17]. High levels of nitrogen significantly altered 26 different metabolites in rice. Transcriptomic analysis showed that eight hub genes were highly correlated with the synthesis of ribosomal and stress proteins [18]. However, a combined transcriptomic and metabolomic analysis of salt tolerance in rice is rarely reported.

The study of AOS in plants is mainly focused on the storage environment of fruits [12, 19, 20], root systems [21, 22], alleviation of stress induced by high temperatures and acid rain [23], disease resistance in fruits [24], and analysis of drought outcomes [15, 25]. AOS can alleviate the decline in plant biomass under salt stress [23]. The effect of AOS on salt tolerance in rice remains unclear. AOS may alleviate the salt stress-induced damage in rice by increasing the levels of antioxidant enzymes and non-enzymatic antioxidants. An intrinsic association between antioxidant genes and metabolites may also exist following salt stress injury.

This study investigates the effect of AOS on the transcriptome and metabolome of rice seedlings including salt-tolerant rice variety FL478 and salt-sensitive rice variety IR29 under salt stress. The molecular mechanism of AOS regulation of salt tolerance in plants has been investigated and the potential practical applications are discussed.

## Results

### Phenotypes of rice seedlings alleviated by AOS under salt stress

**Fig. 1:**
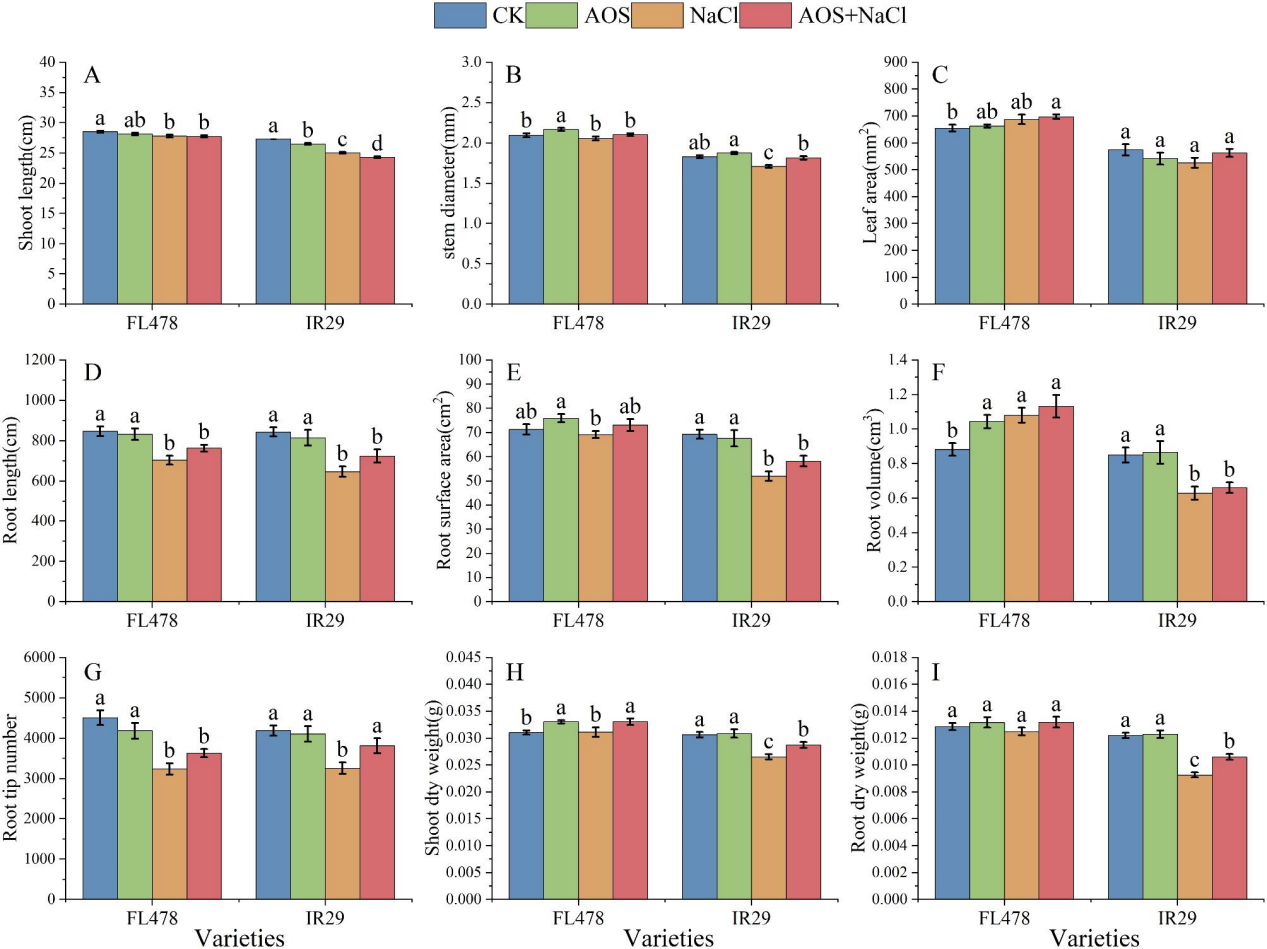
Impact of AOS treatment on phenotypes under salt stress. (**A**) Shoot length. (**B**) Stem diameter. (**C**) Leaf area. (**D**) Root length. (**E**) Root surface. (**F**) Root volume. (**G**) Root tip number. (**H**) Shoot dry weight. (**I**) Root dry weight. Control (CK), alginate oligosaccharides (AOS), salt (NaCl), and alginate oligosaccharides and salt (AOS + NaCl). The shoot phenotype data and dry weight represent the mean of 15 replicates ± SEM. The root phenotype data represent the mean of nine replicates ± SEM. Different letters indicate the importance of several treatments involving the same rice variety

Using potted plants, we investigated the phenotype changes in rice seedings treated with sodium AOS and salts. Compared with NaCl treatment, exposure to a combination of AOS and NaCl alleviated the inhibitory effects of salt stress (Fig. 1).

Salt stress significantly reduced plant height (2.6% and 8.4%) in both varieties. The height of IR29 variety was significantly reduced (2.8%) after AOS treatment (Fig. 1A). Compared with the control, AOS treatment increased the stem diameter in both varieties, with FL478 reaching a significant level (3.5%). Stem diameter decreased under salt stress in both varieties, with IR29 reaching a significant level (6.6%). Compared with salt stress, AOS treatment increased the stem diameter in both varieties, with IR29 reaching a significant level (6.3%) (Fig. 1B). Compared with salt stress, the combination of AOS and NaCl increased the leaf area of FL478 and IR29, but not significantly (Fig. 1C).

NaCl treatment significantly reduced root length (16.9% and 23.4%) and root tip number (28.2% and 22.2%) in both varieties. Combined treatment with AOS and NaCl increased root length and root tip number, which significantly increased the number of root tips (17.1%) in IR29 (Fig. 1D,G). Salt stress reduced the root surface area in both varieties, with IR29 reaching a significant level (25.0%). AOS treatment increased the root surface area but not significantly (Fig. 1E). Compared with the control, NaCl treatment significantly increased the root volume of FL478 (22.5%) and significantly decreased the root volume of IR29 (25.6%). Compared with NaCl treatment alone, the combination of AOS and NaCl increased the root volume of both varieties but not significantly (Fig. 1F).

AOS treatment increased the dry weight of the two rice varieties aboveground and underground but not significantly. Salt stress significantly decreased the aboveground and underground dry weights of rice IR29 (by 13.4% and 23.8%) but not FL478. Compared with NaCl treatment, the combined treatment with AOS and NaCl significantly increased the aboveground and underground dry weights of IR29 by 8.3% and 14.0%, respectively, compared with the control levels (Fig. 1H, I).

### Physiological characteristics of rice seedlings alleviated by AOS under salt stress

**Fig. 2:**
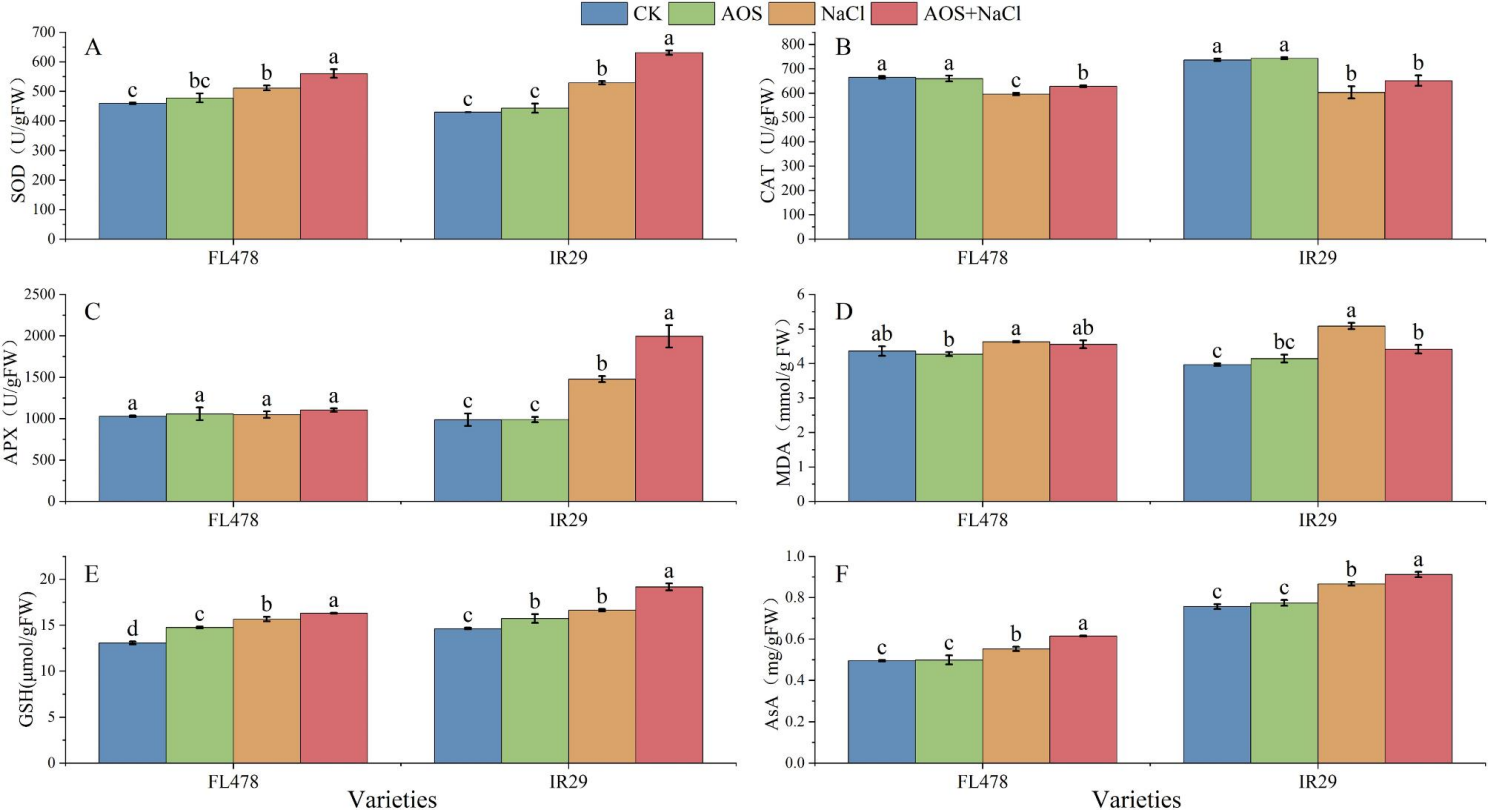
Effects of AOS treatment on physiological characteristics under salt stress. (**A**) SOD activity. (**B**) CAT activity. (**C**) APX activity. (**D**) MDA activity. (**E**) AsA content. (**F**) GSH content. Control (CK), alginate oligosaccharides (AOS), salt (NaCl), and alginate oligosaccharides and salt (AOS + NaCl). The data represent the mean of three replicates ± SEM. Different letters represent the effect of several treatments on the same rice variety

Compared with NaCl treatment, AOS + NaCl treatment reduced the levels of membrane lipid peroxidation, increased the biomass and the antioxidant indices of two rice varieties (Fig. 2). The AOS treatment significantly elevated GSH levels in FL478 and IR29 by 12.9% and 7.5%, respectively (Fig. 2E).

Compared with the control, the ASA activities of FL478 and IR29 under NaCl treatment increased significantly (11.6% and 14.5%, respectively) (Fig. 2F). In contrast, the CAT activities of the two rice varieties were decreased significantly (10.4% and 18.1%, respectively) (Fig. 2B). The SOD and APX activities of the IR29 cultivar were increased significantly (23.1% and 49.7%, respectively) (Fig. 2A, C), and the GSH content of the FL478 cultivar also increased significantly (13.7%) (Fig. 2E). Compared with NaCl treatment, AOS + NaCl treatment significantly increased SOD and CAT activities (9.6% and 5.4%, respectively) and GSH and AsA levels (4.1% and 11.2%, respectively) of the rice variety FL478 (Fig. 2A, B, E, F). IR29 significantly increased SOD and APX activities (by 19.3% and 35.1%, respectively) and GSH and AsA levels (by 15.2% and 14.5%, respectively) (Fig. 2A, C, E, F).

NaCl stress increased the MDA content in the leaves of both rice seedlings and IR29 rice samples significantly (28.3%). Compared with NaCl treatment, AOS + NaCl treatment reduced the MDA content of both varieties, and significantly decreased the levels in the rice variety IR29 (13.25%).

The AOS reduced the effect of salt stress on rice by increasing SOD activity and AsA and GSH levels. Nonetheless, the effect of AOS on the antioxidant levels of the two rice varieties differed. Exogenous AOS application increased the CAT levels of FL478 to offset the effects of salt stress. AOS improved salt tolerance by increasing the APX content, and thereby reducing membrane lipid peroxidation.

### Transcriptomic response of rice seedlings exposed to salt stress and sodium alginate oligosaccharide treatment

RNA sequences derived from 18 samples were analyzed (Table S1). Each sample yielded an average size of approximately 6.68 GB of bases. The average Q30 was 93.47%. The sequencing quality and library construction of reaction samples were good, and the data accuracy was high. Each sample was a clean read against a reference genome sequence. The mapping ratio was 89.91–91.17%, and the unique mapping ratio was 87.55-88.87%. These data met the criteria for subsequent analysis, and the correlation coefficient of the three replicates in each treatment above was 0.9 (Figure S1).

The number of differentially expressed genes was analyzed via transcriptome sequencing. A p < 0.05 and Log2FC ≥ 1 were used to screen differentially expressed genes. Compared with CK, 732 (483 upregulated and 249 downregulated) and 305 (169 downregulated and 136 downregulated) differentially expressed genes were identified in samples treated with NaCl and AOS + NaCl, respectively. A total of 662 differentially expressed IR29 genes (272 upregulated, 390 downregulated) and 1030 (307 upregulated, 723 downregulated) were identified in samples treated with NaCl and AOS + NaCl, respectively. A total of 1015 differentially expressed genes were detected in FL478 and IR29 varieties treated with NaCl + AOS (567 upregulated and 448 downregulated) and 225 (41 upregulated and 184 downregulated), respectively, compared with NaCl treatment alone (Table 1). RT-qPCR was used to detect the expression levels of eight identified transcriptome (RNA-ref) differential genes to validate the RNA-ref data (Figure S2). The results of RT-qPCR were positively correlated with those of RNA-ref.

**Table 1:** Number of differentially expressed genes in rice seedlings

Combinations	Up-regulation	Down-regulation	All DEGs
FLCK-vs-FLNaCl	483	249	732
FLCK-vs-FLNaCl + AOS	169	136	305
FLNaCl-vs-FLNaCl + AOS	567	448	1015
IRCK-vs-IRNaCl	272	390	662
IRCK-vs-IRNaCl + AOS	307	723	1030
IRNaCl-vs-IRNaCl + AOS	41	184	225

**Fig. 3:**
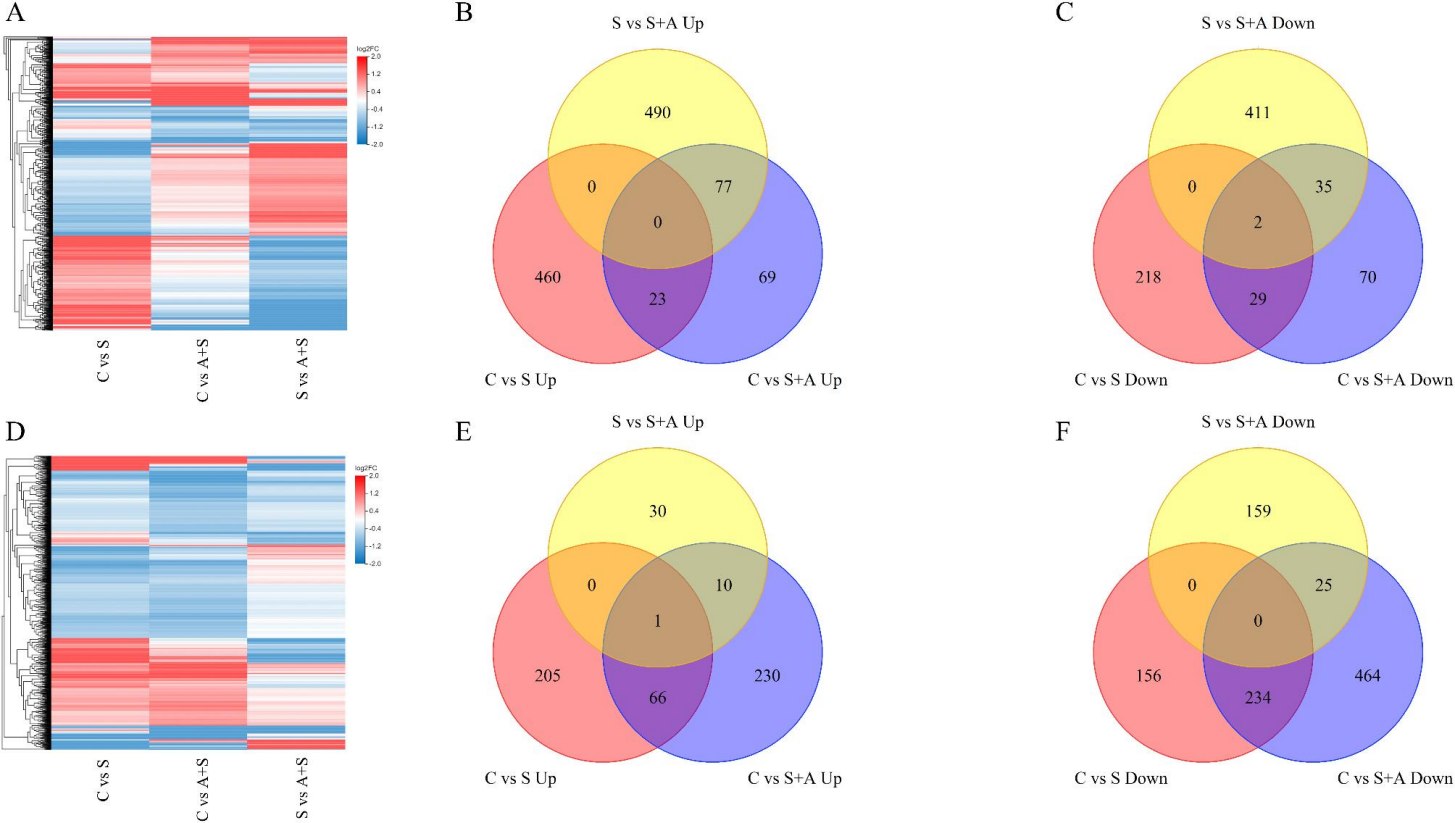
Cluster heat map and Venn diagram of differentially expressed genes in FL478 (**A**) and IR29 (**D**) under different treatments. FL478 seedlings: Venn Diagram of upregulated genes (**B**) and Venn Diagram showing downregulated genes (**C**) under different treatments. IR29 rice seedlings: Venn Diagram of upregulated (**E**) and downregulated genes (**F**) under different treatments. Contrast treatments included S, salt treatment; A + S, alginate oligosaccharides + salt treatment. Differential genes were determined using Log2FC > = 1 and P < 0.05

Table 1; Fig. 3 show the number of downregulated genes in rice seedlings under different treatments, revealing the expression patterns of FL478 and IR29 in response to salt, AOS, and AOS combined with salt treatments. A higher number of differentially expressed genes were identified in FL478 treated with NaCl than in FL478 treated with AOS + NaCl compared with CK, with a higher number of upregulated genes in FL478 treated with NaCl than AOS + NaCl. In IR29, a higher number of differentially expressed genes were expressed following treatment with AOS + NaCl than in IR29 treated with NaCl alone, with a higher number of downregulated genes in samples treated with NaCl than in those treated with AOS + NaCl. These results indicate that the two rice varieties had different regulatory pathways associated with salt tolerance and response to AOS.

Cluster analysis revealed that AOS and salts significantly changed the differential gene expression in rice (Fig. 3A, D). Further analysis using Venn diagrams showed fewer overlapping genes in FL478 and IR29 varieties exposed to NaCl compared with control and those treated with NaCl compared with AOS + NaCl (Fig. 3). These results indicated that rice plants responded to salt stress and AOS by expressing different genes.

### AOS regulate functional genes to alleviate salt stress in rice

NaCl and AOS + NaCl treatments significantly affected gene expression in both rice varieties. In FL478/IR29 rice seedlings, the specific differential genes upregulated and downregulated by CK vs. AOS + NaCl were 146/240 and 105/489, respectively. The significant effect of exogenous AOS on the gene expression of rice plants under salt conditions is presented schematically in Fig. 3.

**Fig. 4:**
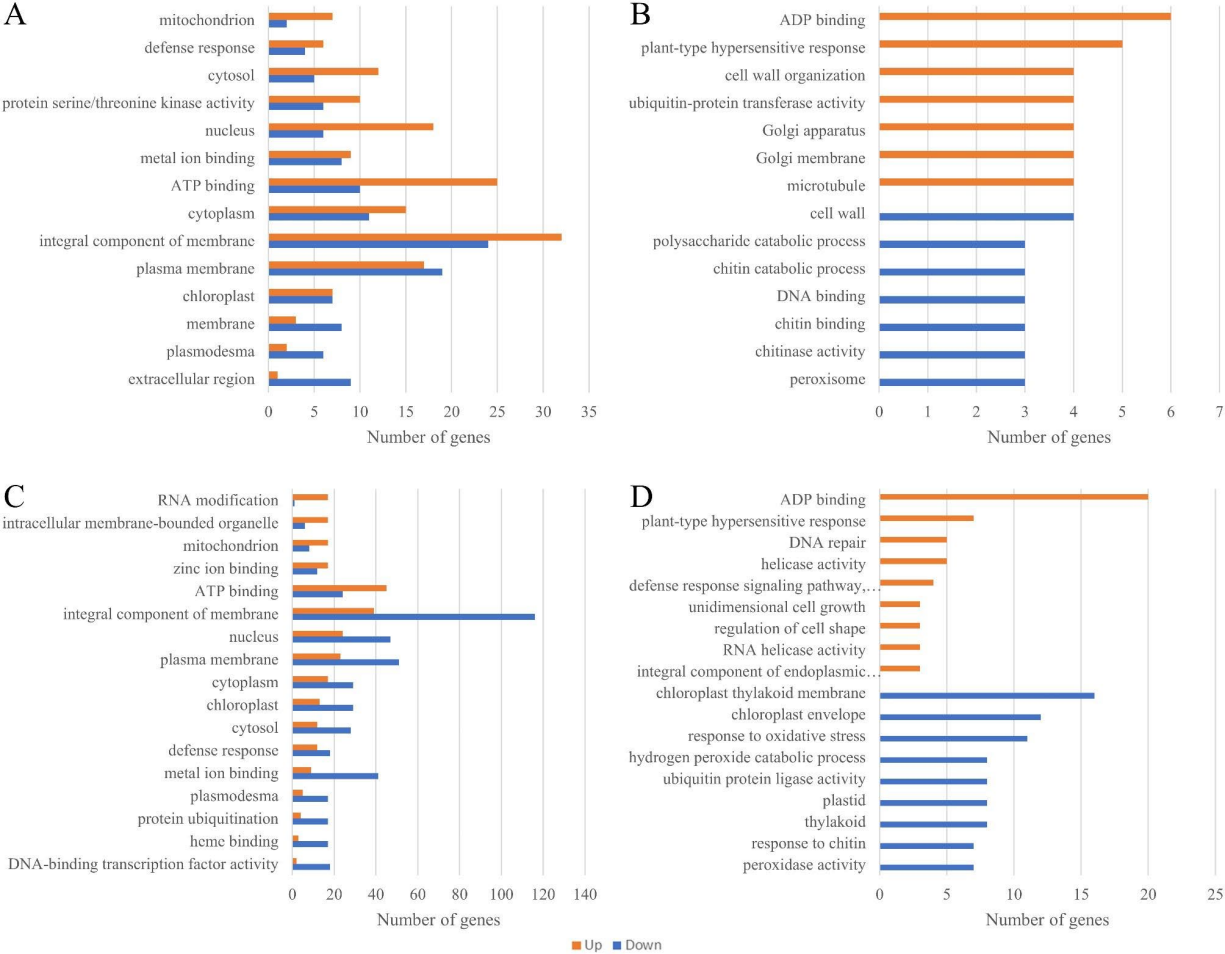
GO enrichment analysis of upregulated or downregulated genes in rice seedlings treated with AOS and salt. FL478: GO enrichment classes of upregulated and downregulated genes (**A**) and upregulated and downregulated unique GO enrichment classes (**B**). IR29: GO enrichment classes of upregulated and downregulated genes (**C**) and upregulated and downregulated unique GO enrichment classes (**D**). The X-axis represents the number of differential genes and the Y-axis GO enrichment annotation

Based on GO enrichment analysis of differential genes, the most common types of upregulated and downregulated genes in FL478 rice seedlings were associated with integral components of membranes (GO:0016021), plasma membranes (GO:0005886), ATP binding (GO:0005524), cytoplasmic (GO:0005737) and nuclear components (GO:0005634) (Fig. 4A). Among the upregulated and downregulated genes in IR rice seedlings, the primary categories were: integral components of membranes (GO:0016021), plasma membranes (GO:0005886), nucleus (GO:0005634), ATP binding (GO:0005524), and metal ion-binding targets (GO:0046872) (Fig. 4C).

Among 410 upregulated genes in FL478 rice seedlings, ADP binding (GO:0043531) and plant-type hypersensitive genes were the most enriched responses (GO:0009626), with additional categories of microtubule (GO:0005874), Golgi membrane (GO:0000139), Golgi apparatus (GO:0005794), ubiquitin-protein transferase activity (GO:0004842), and cell wall organization (GO:0071555). Among the 490 downregulated genes, the GO-rich types were cell wall (GO:0005618), peroxisome (GO:0005777), chitinase activity (GO:0004568), chitin binding (GO:0008061), DNA binding (GO:0003677), chitin catabolim (GO:0006032), and polysaccharide catabolism (GO:0000272) (Fig. 4B).

The 139 upregulated genes in IR29 rice seedlings included the most enriched category of GO:0043531 (ADP binding), GO:0009626 (plant-type hypersensitive response), GO:0004386 (helicase) activity), GO:0006281 (DNA repair), and GO:0009870 (defense response signaling pathway, resistance gene-dependent). Among the downregulated genes, the dominant GO categories included GO:0009535 (chloroplast thylakoid membrane), GO:0009941 (chloroplast envelope), GO:0006979 (response to oxidative stress), GO:0009579 (thylakoid), GO:0009536 (plastid), GO:0061630 (ubiquitin protein ligase) activity), and GO:0042744(peroxide catabolic process), as shown in Fig. 4D.

### Gene expression in rice seedlings under salt stress induced by AOS

Compared with the control treatment, some genes were differentially expressed only under the AOS + salt treatment. In FL478, 13 photosynthetic, 5 Serine/threonine kinases, 9 cell wall, 6 carbohydrate, and 12 plant hormone-related genes were significantly different compared with the control data. The upregulated genes and IR29 expression levels under salt stress induced by sodium AOS treatment correlated with 22 photosynthetic, 7 carbohydrate, and 11 serine/threonine kinase-related genes, as well as 15 plant hormones, 18 salt response and 24 transcription factors (Table S3). Among them, *OsCAB1*, *OsLhcb2.1*, *OsLhcb6*, *OsLHCB4* and *OsLhcp* participated in the regulation of photosystem II, whereas *OsPMEI28* mediated the regulation of monosaccharide and lignin content in stems. *OsGA2ox6* participated in the regulation of gibberellin, and *OsRLCK106* was expressed in response to salt stress in rice. These findings indicate that sodium AOS play an active role in regulating salt tolerance in rice through multiple pathways. The photosynthetic components (OsPORA, OsDIMT2) are related to identical genes in the two varieties. In contrast, other differentially expressed genes in the two varieties indicate that AOS may exhibit different transcriptional regulation modes in FL478 and IR29.

### Changes in the metabolites of rice seedlings treated with AOS under salt stress

**Fig. 5:**
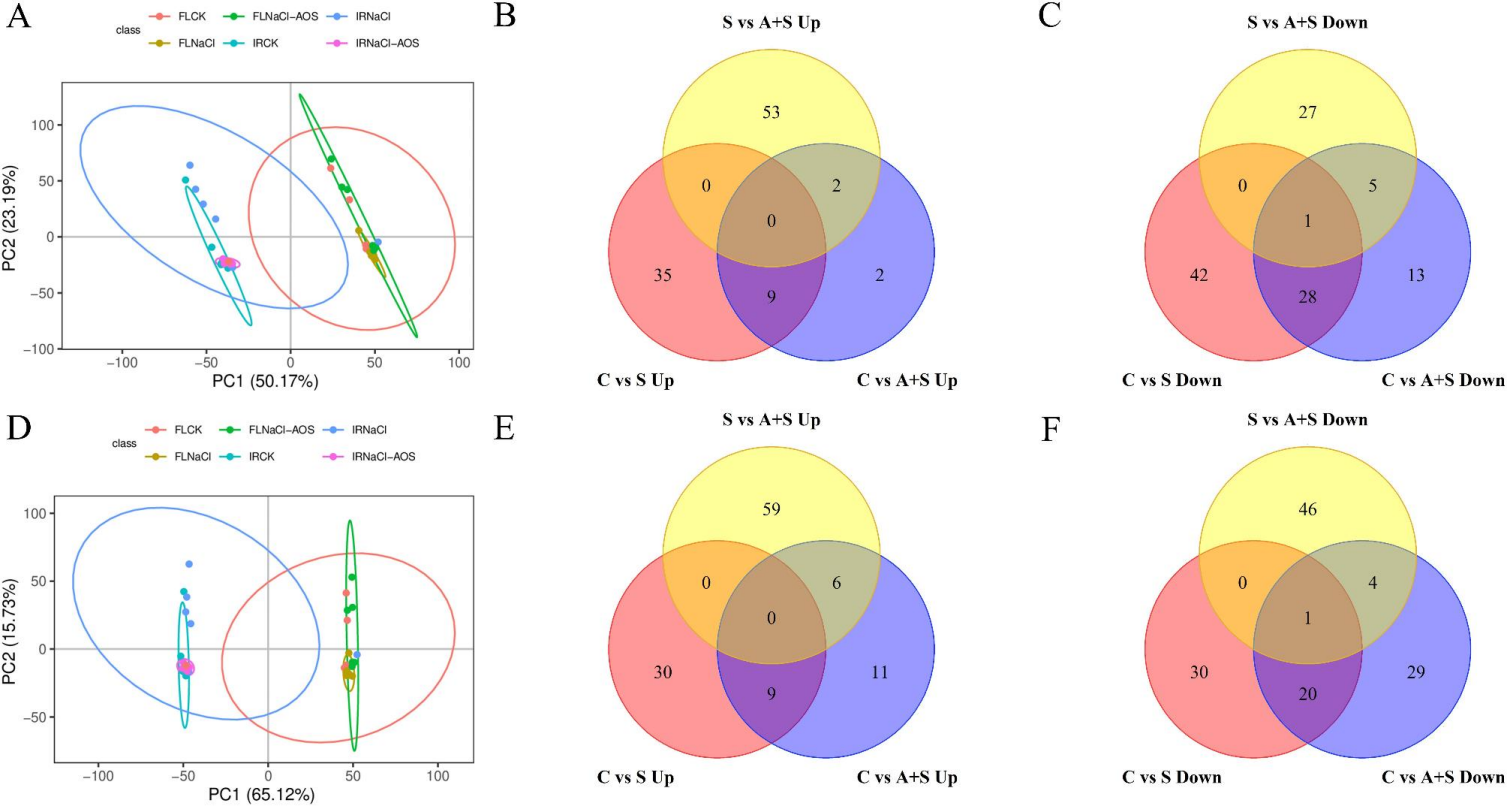
Principal component analysis (PCA) of differentially expressed metabolites following different treatments of FL478 and IR29 and Venn diagrams of metabolites in different groups. Positive ion mode PCA (**A**). Negative ion mode PCA (**D**). FL478: Venn diagram showing increased and decreased metabolite abundance under different treatments (**B** and **C**). IR29: Venn diagram of increased and decreased metabolite abundance under different treatments (**E** and **F**). C. contrast treatment; S, salt treatment; A + S, alginate oligosaccharides + salt treatment. Log2FC > = 1 and P < 0.05

To evaluate the effects of exogenous AOS treatment on the metabolic variations between FL478 and IR29 rice seedlings under salt stress, the differential metabolites were screened using the filters VIP ≥ 1, fold change ≥ 1.2 or ≤ 0.83, and a p-value < 0.05. FL478 and IR29 samples treated with CK, S and A + S were compared. Two principal components (PC1 = 50.17%, PC2 = 23.19%) in positive ion mode and two principal components (PC1 = 65.12%, PC2 = 15.73%) in negative ion mode were obtained using PCA analysis, with differences between the two cultivars in both modes (see Fig. 5A, D). A total of 851 metabolites were identified in all samples under both modes. Among them, 549 differential metabolites (VIP ≥ 1, fold change ≥ 1.2 or ≤ 0.83, p-value < 0.05) were significantly abundant in at least one group (Table 2).

**Table 2:** Breakdown of differential metabolites for both rice species

Group	Total	Up	Down
FLCK-vs-FLNaCl	115	44	71
FLCK-vs-FLNaCl + AOS	60	13	47
FLNaCl-vs-FLNaCl + AOS	88	55	33
IRCK-vs-IRNaCl	90	39	51
IRCK-vs-IRNaCl + AOS	80	26	54
IRNaCl-vs-IRNaCl + AOS	116	65	51

The levels of 44 metabolites increased, while the levels of 71 decreased in salt-treated FL478 rice seedlings compared with control seedlings. The abundance of 39 and 51 metabolites in IR29 rice seedlings increased and decreased, respectively. Treatment with AOS combined with salt increased the metabolite abundance of FL478 rice seedlings by 13 and decreased by 47 compared with the control samples. In IR29 rice seedlings, the metabolite abundance increased and decreased by 26 and 54 species, respectively, compared with the control data (Table 2). The differential metabolites of rice after treatment were analyzed using Venn diagrams. The treatment with AOS combined with salt increased the abundance of 4 and 17 different metabolites in the FL478 and IR29 rice seedlings, while decreasing the abundance of 18 and 33 different metabolites, respectively (Fig. 5B, C, E, F). Therefore, the differences in metabolite expression induced by AOS in the two rice varieties changed significantly under salt stress. In FL478, the abundance of flavonoids (vicenin II, wedelolactone), indole and indole derivatives (6-hydroxymelatonin), and gamma butyrolactones (dehydroascorbic acid) increased significantly. Metabolites such as carbohydrates (D-(+)-glucosamine), terpenoids (2,6-di-tert-butyl-1,4-benzoquinone), fatty acyls (10-nitrolinoleate) was significantly decreased. Amino acids (L-histidine, aspartate, L-norleucine, N-acetyl-L-methionine), terpenoids (rehmannioside C and andrographolide) and other differentially expressed metabolites were significantly increased in abundance in IR29. The abundance of metabolites such as rehmannioside C and andrographolide increased significantly in IR29. Flavonoids (neomangiferin 5,7-dihydroxychromone, vitexin, and wedelolactone), polyketides [PK] (dodecyl sulfate, and 6-gingerol) and others were significantly reduced in IR29 (Table S4). Among them, 6-hydroxymelatonin, wedelolactone, and L-histidine play an active role in the antioxidant mechanism.

**Table 3:** Differential expression of metabolites with significant upregulation or downregulation in FL478 treated with alginate oligosaccharides plus salt treatment compared with levels in the control samples

Metabolite ID	Metabolite Name	P-value	VIP	Log_2_FC
15.16_486.26264	Andrastin A	0.00	1.53	-0.33
0.844_174.01649	Dehydroascorbic acid	0.00	1.87	0.64
17.37_350.16205	7-(2,3-Dimethylphenyl)-2-methoxy-2,3-dihydro-1 H-pyrrolo[2,1-c] [1, 4]benzodiazepine-5,11(10 H,11aH)-dione	0.00	1.69	-0.57
1.642_297.10731	N(6)-oh-me-adenosine	0.00	1.75	-0.54
15.159_220.14657	2,6-Di-tert-butyl-1,4-benzoquinone	0.01	1.64	-0.36
9.905_314.04186	Wedelolactone	0.01	1.25	0.35
17.766_354.19332	2-(3,4-Dimethoxyphenyl)-N-(2-piperidinophenyl)acetamide	0.01	1.84	-0.82
11.85_478.25486	Diflucortolone pivalate	0.01	2.43	-0.75
9.363_307.21470	10-Nitrolinoleate	0.01	2.76	-2.95
2.302_248.11946	6-Hydroxymelatonin	0.01	1.87	0.86
1.073_179.07961	D-(+)-Glucosamine	0.02	1.50	-0.50
7.915_467.22892	1-{[(1s,4s,6s)-6-isopropyl-3-methyl-4-{[5-(4-pyridinyl)-1,3,4-oxadiazol-2-yl]methyl}-2-cyclohexen-1-yl]methyl}-3-phenylurea	0.02	2.43	-2.77
7.196_397.14212	Methyl 2,7,7-trimethyl-5-oxo-4-[3-(2-thienyl)-1 H-pyrazol-4-yl]-1,4,5,6,7,8-hexahydro-3-quinoline carboxylate	0.02	1.43	-0.36
5.726_594.15930	Vicenin II	0.02	1.22	0.38
11.851_330.18063	(1r,4as)-7-(2-hydroxypropan-2-yl)-1,4a-dimethyl-9-oxo-3,4,10,10a-tetrahydro-2 h-phenanthrene-1-carboxylic acid	0.03	1.59	-1.24
8.181_467.22889	N-(3,4-dimethoxybenzyl)-2-[(3r,4s)-3-{[5-(4-fluorophenyl)-1,2-oxazol-3-yl]methyl}-4-piperidinyl]acetamide	0.03	1.56	-1.27
10.031_490.27684	N-({(2R,4 S,5R)-5-[3-(3,4-Dimethoxyphenyl)-1-methyl-1 H-pyrazol-5-yl]-1-azabicyclo[2.2.2]oct-2-yl}methyl)-2-ethylbutanamide	0.04	1.14	-0.45
13.967_334.21199	(3E)-3-(Hydroxymethyl)-2-oxo-5-[(1 S,8aS)-5,5,8a-trimethyl-2-methylenedecahydro-1-naphthalenyl]-3-pentanoic acid	0.04	1.02	-0.40
16.124_382.27154	1a,1b-Dihomo prostaglandin f2О±	0.04	1.17	-0.93
10.011_472.26648	4-Cyano-n-({(1s,4s,6s)-6-isopropyl-3-methyl-4-[2-(4-methyl-1-piperazinyl)-2-oxoethyl]-2-cyclohexen-1-yl}methyl)benzamide	0.04	2.00	-0.71
8.793_376.20933	3-[4-(tert-Butyl)anilino]-1-[4-(tert-butyl)phenyl]-2,5-dihydro-1 H-pyrrole-2,5-dione	0.05	1.12	-0.98
7.725_528.18623	1,4:3,6-Dianhydro-2-[(benzylsulfonyl)amino]-5-{[4-(4-biphenylyl)-2-pyrimidinyl]amino}-2,5-dideoxy-L-iditol	0.05	1.62	-2.80

A Kyoto Encyclopedia of Genes and Genomes (KEGG) analysis of the metabolites was used to investigate the primary metabolic pathways modified by AOS treatment of FL478 and IR29 rice seedlings under salt stress. FL478 and IR29 were annotated into 4 and 16 KEGG pathways for samples treated with AOS combined with salt. The co-enriched FL and IR pathways included ascorbate and aldarate metabolism (ko00053) and glutathione metabolism (osa00480). The unique metabolic enrichment pathways in FL478 include amino sugar and nucleotide sugar metabolism (ko00520) and tryptophan metabolism (ko00380). The unique metabolic pathways in IR29 include histidine metabolism (ko00340), beta-alanine metabolism (ko00410), stilbenoid, diarylheptanoid and gingerol biosynthesis (ko00945), flavonoid biosynthesis (ko00941), aminoacyl-tRNA biosynthesis (ko00970), and biosynthesis of secondary metabolites (ko01110) (Table S5).

### Combined transcriptomic and metabolomic analysis

**Fig. 6:**
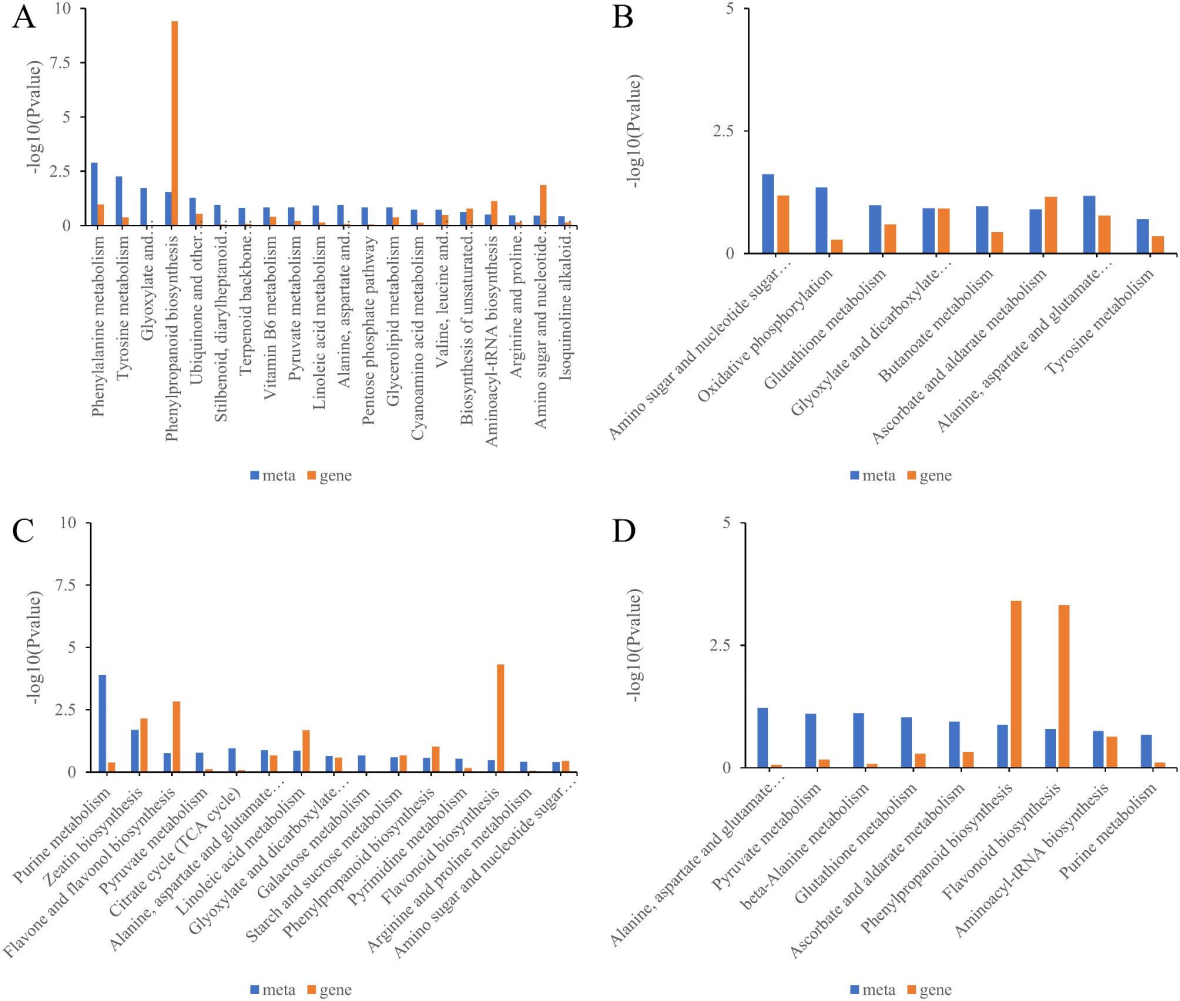
Differential expression of regulatory genes and metabolites mapped to KEGG pathways in control vs. salt treatment groups and control vs. sodium alginate oligosaccharide + salt treatment group. FL478: (**A**) Enrichment pathway of salt treatment, and (**B**) Enrichment pathway of alginate oligosaccharides + salt treatment. IR29: (**C**) Enrichment pathway of salt treatment, and (**D**) Enrichment pathway of alginate oligosaccharides + salt treatment

**Fig. 7:**
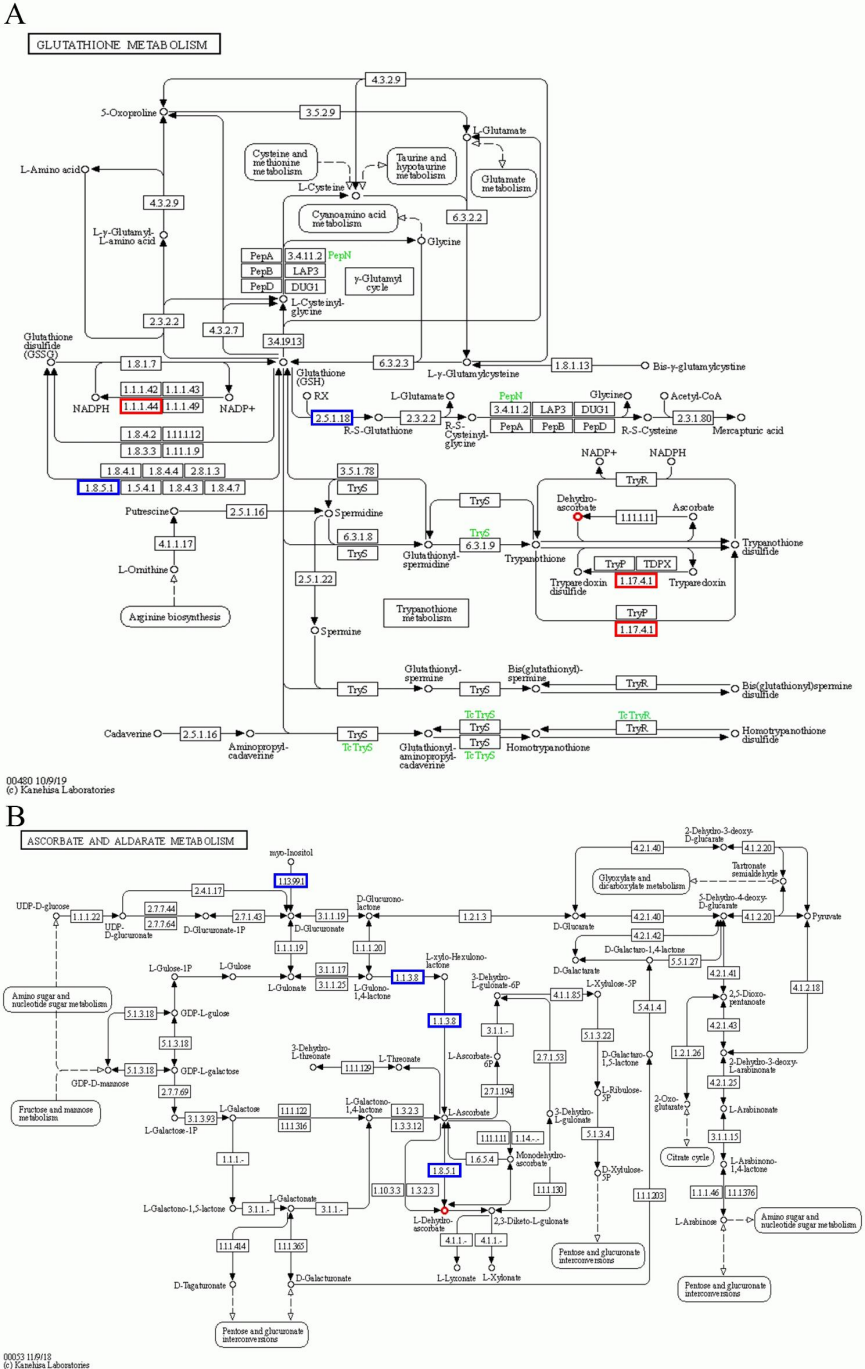
Enrichment pathway of genes and metabolites in FL478 under salt stress mediated by alginate oligosaccharides. Glutathione metabolic pathway (**A**). Ascorbic acid and erythrosine metabolic pathway (**B**). The red highlighted areas in A and B represent significantly upregulated genes or metabolite levels. Blue represents significantly downregulated genes or metabolites with reduced abundance. Used with permission of Kanehisa Laboratories

**Fig. 8:**
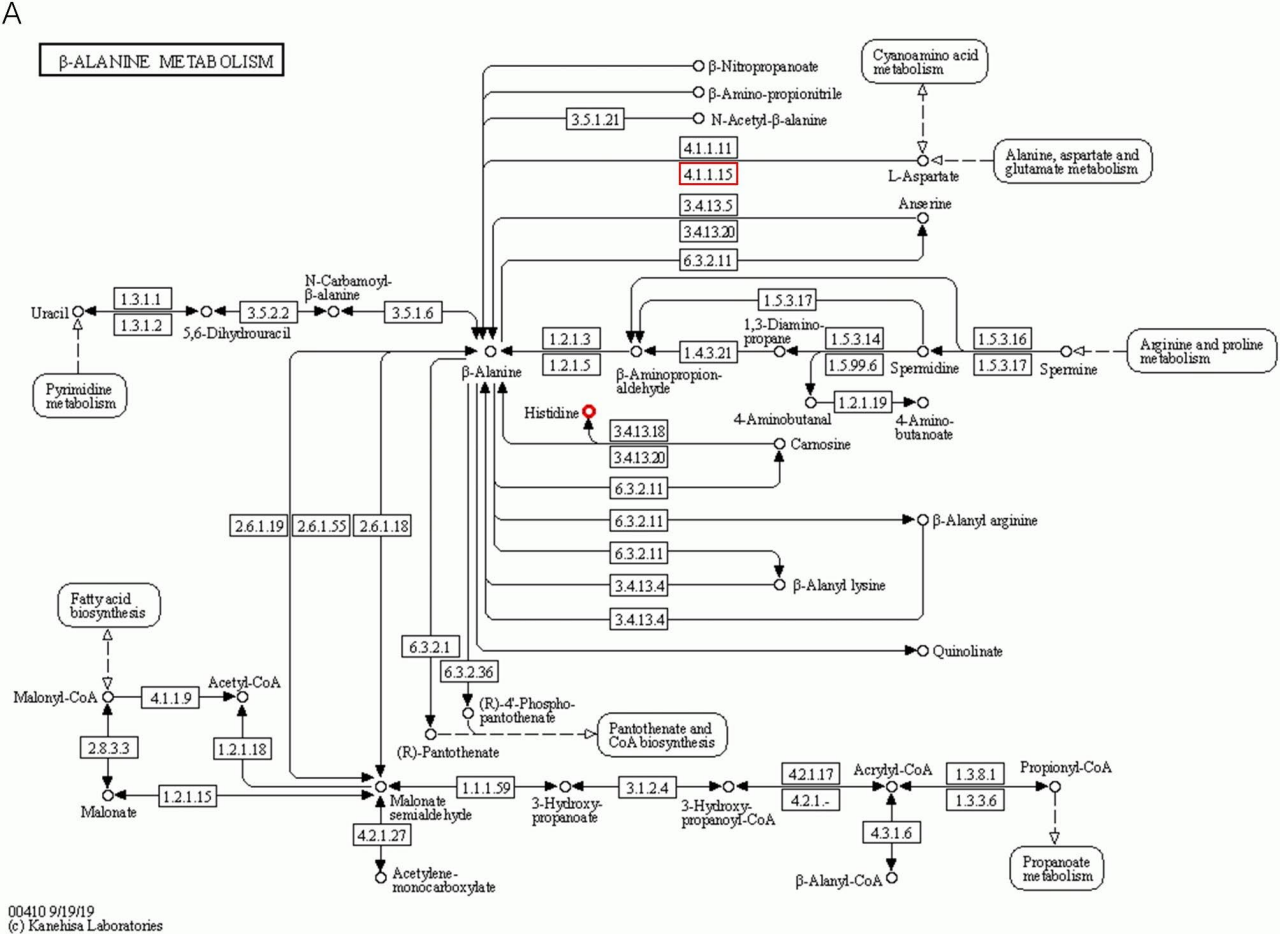
Alginate oligosaccharides contribute to beta-alanine metabolism enrichment pathway in IR29 under salt stress. Red highlights indicate significantly upregulated genes or metabolites with a significant increase in abundance. Used with permission of Kanehisa Laboratories

To explore the effects of AOS on genes and metabolites in rice cultivated under salt stress, differential genes and metabolites in control group and groups treated with salt as well as in control and AOS + salt combined were simultaneously enriched in KEGG pathways. Twenty and eight KEGG pathways were identified in FL478 following salt exposure and treatment with AOS and salt combined, while 15 and nine KEGG pathways were identified in IR29 exposed to salt and AOS combined with salt (Fig. 6). Glutathione metabolism (ko00480), ascorbate and aldarate metabolism (ko00053) were the key pathways enriched in FL478. The pathways for beta-alanine metabolism (ko00410), glutathione metabolism (ko00480), ascorbate and aldarate metabolism (ko00053), and aminoacyl-tRNA biosynthesis (ko00230) were significantly enriched in IR29 only under AOS plus salt treatment. Among them, glutathione metabolism (ko00480), ascorbic acid and aldarate metabolism (ko00053) were significantly enriched in FL478 and IR29 varieties (Fig. 7). Dehydroascorbic acid was simultaneously enriched in both pathways. Histidine abundance increased significantly following sodium AOS treatment under salt stress. The related gene GAD3 was also significantly upregulated (Fig. 8). AOS may induce the GAD3 gene to modulate histidine metabolism.

**Table 4:** Co-enrichment pathways of transcriptome and metabolome in rice seedlings treated with alginate oligosaccharides + salt

Varieties	Pathway	Metabolite ID	Metabolite Name	logFC
FL478	Glutathione metabolism (ko00480)	0.844_174.01649	Dehydroascorbic acid	0.64
	Ascorbate and aldarate metabolism (ko00053)	0.844_174.01649	Dehydroascorbic acid	0.64
IR29	beta-Alanine metabolism (ko00410)	0.771_155.06943	L-Histidine	0.35
	Glutathione metabolism (ko00480)	0.844_174.01649	Dehydroascorbic acid	-0.41
	Ascorbate and aldarate metabolism (ko00053)	0.844_174.01649	Dehydroascorbic acid	-0.41
	Aminoacyl-tRNA biosynthesis (ko00970)	0.771_155.06943	L-Histidine	0.35

## Discussion

AOS represent the degradation products of alginate, which promote growth, improve plant resistance to abiotic stress, activate immunity and extend fruit storage time [26]. This study investigated the effects of spraying AOS on the phenotype, physiological characteristics, biomass accumulation, transcriptome and metabolome of rice leaves. The results showed that salt stress elevated MDA content, plant height, root length, number of root tips, IR29 root surface area, root volume, and stem basal width. The aboveground and belowground dry weight of the two rice varieties were significantly reduced under salt stress (Figs. 1 and 2). Under salt stress, the MDA levels in FL478 and IR29 were significantly reduced, while the root tip number, root length, and biomass were significantly increased under AOS plus salt treatment (Figs. 1 and 2). Salt stress was substantially higher in salt-sensitive rice than in salt-tolerant rice. These results indicated that AOS dramatically alleviated the damage induced by salt stress. Substantial levels of SOD and APX were generated in the antioxidant system of rice following salt treatment (Fig. 2), which was consistent with the results of Lee et al., 2001. Under salt stress, AOS treatment significantly increased the levels of non-enzymatic antioxidants ASA and GSH together with the activities of antioxidant enzymes SOD, CAT and APX in two rice varieties, FL478 and IR29 (Fig. 2). Analysis of transcriptome data in IR29 also revealed that genes related to salt stress response were highly expressed (Table S3). These studies indicated that AOS reduce the oxidative damage caused by salt stress in rice by inducing the expression of salt-stress-related genes and increasing the levels of antioxidant enzymes and non-enzymatic antioxidants.

Salt stress can inhibit photosynthesis by altering enzyme activity, damaging chloroplasts, restricting electron flow, and triggering reactive oxygen species [27]. It is crucial to enhance plant photosynthesis during salt stress. Previous studies have shown that exogenous melatonin (MT) (F. Yan et al., 2021) and glycine betaine (GB) [28], which act as plant growth regulators, alleviate salt stress-induced damage by improving photosynthesis in rice. This study shows that AOS induce differential expression of 13 and 22 photosynthesis-related genes in FL478 and IR29 under salt stress (Table S3). *OsCAB1* [29] and the genes regulating LHCB proteins(*OsLhcb2.1, OsLhcb6, OsLHCB4*) [30, 31] and *OsLhcp* [32] were involved in the regulation of light trapping in photosystem II. AOS may alleviate stress damage by modulating light-trapping proteins in rice seedlings exposed to salt stress. In wheat, LHCs are highly expressed in chloroplast tissues, and overexpression often improves tolerance to stress [33]. However, in this experiment, the AOS downregulated the light-harvesting protein-related genes. AOS may have complex effects on rice photosynthesis under salt stress, which requires further experimental investigation.

Plants adapt to environmental conditions by changing cell wall structure and composition under abiotic stress [34, 35]. Wang’s study found that garlic differentially expressed cell wall-and hormone-related genes under salt stress, suggesting that plants adapt to salt stress by changing the content and structure of plant cell wall components. AOS induced the differential expression of six carbohydrate, nine cell wall, and 12 hormone-related genes in FL478 rice seedlings under salt stress (Table S3). Among them, OsPMEI28 differentially altered the methyl ester groups, monosaccharides, and lignin levels in the plant stem [36]. *CHT10* controls the synthesis of OsGA2ox6 chitin [37]. Other genes control the synthesis of gibberellin and other plant hormones [38]. Previous studies also reported that exogenous compounds regulate cell wall composition and other factors, such as carbon monoxide, increased the content of pectin and hemicellulose in cell walls [39]. The current study findings suggest that AOS may alleviate salt stress damage by regulating material metabolism and the structure of cell walls under salt stress. Due to experimental limitations, the cell wall structure is less studied [34]. However, the regulation mechanism of AOS in cell walls is unclear. Further studies are required to investigate AOS regulation in the cell wall components of rice seedlings under salt stress.

Receptor-like kinases (RLK) expressed in plant signal transduction pathways are usually composed of extracellular receptor domains and intracellular Ser/Thr kinase domains that transmit signals by phosphorylating target proteins, thereby coordinating the expression of mechanogene-related genes under salt stress [40, 41]. The current study uncovered many serine/threonine kinase genes, which were highly expressed in both rice species. Among them, the receptor-like cytoplasmic kinase (RLCK) gene OsRLCK106 in FL478 and the RLK genes OsRLCK150 and OsRLCK295 in IR29 were highly upregulated under salt stress mediated by AOS (Table S3). RLCK, an RLK, has been shown to confer tolerance to abiotic stress in plants [42]. Previous studies have shown that rice can upregulate OsRLCK106 in response to salt stress [43]. In this study, AOS were found to play a crucial role in signal transduction in response to salt stress and regulation of ROS homeostasis by regulating Ser/Thr kinase-related genes [40].

Analyses of differential metabolites suggest that the metabolite levels of FL478 and IR29 rice seedlings induced by AOS were significantly changed under salt stress. Levels of 6-hydroxymelatonin, wedelolactone and L-histidine in IR29 were significantly increased in FL478 (Table 3, S4). These metabolites have been reported to be involved in antioxidant processes [44–46], suggesting a positive role in AOS-mediated stress response. The joint analysis also found that L-histidine and GAD3 were simultaneously enriched in the beta-alanine metabolism pathway [47] only when AOS and salt were introduced (Fig. 8). GAD3 may play an important role in L-histidine metabolism mediated by AOS under salt stress. Other metabolites such as vicenin II, wedelolactone, andrographolide, rehmannioside C, L-norleucine and N-acetyl-L-methionine, also increased under salt stress mediated by AOS. These metabolites may positively affect salt stress mediated by alginate oligosaccharides.

The glutathione ascorbic acid cycle (AsA-GSH) resists the effects of stress by scavenging free radicals and reducing peroxides in plants [48]. Combined transcriptomic and metabolome analysis shows that sodium AOS treatment was significantly enriched in glutathione metabolism [49] and disinhibitory thrombotic metabolism [50] pathways under salt stress, and the metabolite dehydroascorbic acid (DHA) was enhanced substantially in both pathways simultaneously (Table 4; Fig. 7). DHA can be easily transported than ascorbic acid (AA) and is better absorbed in plant membranes than AA [51]. Upon entry into the cell, the AA is rapidly catalyzed by dehydroascorbate reductase [52]. Thus, DHA may play an important role in the ASA-GSH cycle of alginate-oligosaccharide-mediated salt stress. Notably, ascorbic acid and glutathione levels were significantly increased by AOS under salt stress, especially in IR29 (Fig. 2). Ascorbic acid and glutathione, important non-enzymatic antioxidants in the AsA-GSH cycle, protect plants from oxidative stress [53]. Therefore, the outcomes of the current study suggest that salt stress induces the activation of the AsA-GSH cycling pathway by AOS to alleviate oxidative damage by regulating DHA metabolism.

As a plant growth regulator, the potential application of AOS in agriculture mainly depends on their regulatory effect. Any large-scale applications should be based on economic feasibility and environmental factors. AOS can effectively reduce plant salt stress-induced damage and inhibition. Further, exogenous AOS regulate a variety of pathways in plants. AOS is an oligosaccharide with high solubility and is easily biodegraded with minimal environmental impact [54, 55]. Small doses of AOS can be used to regulate growth effectively. Therefore, AOS represent environmentally friendly agents with potential for practical application.

## Conclusions

Salt stress decreases plant height, stem diameter, root length, root tip number, root surface area, and rice biomass, and increases membrane lipid peroxidation This study provides an excellent validation of exogenous AOS to improve the tolerance of rice to salt stress. Exogenous AOS can improve the salt tolerance of rice by regulating signal transduction, modulating the light-trapping process of photosystem II, modifying cell wall formation and various antioxidant pathways, and regulating the balance of antioxidant metabolites. These advantages are sufficient to consider the commercial application of AOS in rice agriculture. Our results elucidate the intrinsic molecular mechanism of the effect of sodium AOS on salt-stressed rice plants. This study provides a framework and valuable empirical data for further investigation into the relationship between sodium AOS treatment and plant abiotic stress. Regulatory pathways should be studied in depth to elucidate potential mechanisms to generalize these findings.

## Materials and methods

### Plant growth and treatment

The rice salt-tolerant variety FL478 and salt-sensitive variety IR29 were used in this investigation. The seeds were provided by Germplasm Bank of the College of Coastal Agriculture Sciences, Guangdong Ocean University. After disinfection with 2.5% NaClO for 15 min, the seeds were washed with distilled water, soaked in water under 30℃ and in darkness for 24 h. The experiments were performed in 2021 at the Binhai Agricultural College of Guangdong Ocean University greenhouse (under natural light, and alternating day and night temperatures 25/20 ± 2 °C, and a relative humidity of 60%) with potted plants. The nutrient pot base was a mixture of red soil and sand in a ratio of 3:1. Each pot weighed 2.5 kg (210 mm straight on the upper bottom, 160 mm straight on the lower bottom, and 180 mm high). Seventy-five seeds were sown in each pot, cultured to one leaf and one heart stage (6 days after sowing), and treated with NaCl and AOS (provided by the Dalian Institute of Chemical Physics, Chinese Academy of Sciences), Sodium alginate was obtained by decreasing hydrolysis under the action of lyase, which was generated by β-D-mannuronic acid and α-L-guluronic acid connected by β-1-4 glycosidic bonds. The molecular formula of sodium alginate is (C6H7O6Na)n; its degree of polymerization is 2–10, with a uronic acid composition of M/G = 7:3 a uronic acid content > 90%). Either distilled water or 900 mg·L^− 1^ AOS were sprayed evenly on both sides of each leaf. Moist and non-dripping treatment is recommended. Either 800 mL water or 0.3% NaCl solution was used for treatment in addition to other treatment groups: distilled water + clear water (CK), sodium alginate oligosaccharide + clear water (AOS), distilled water + NaCl solution (NaCl), or sodium alginate oligosaccharide + NaCl solution (AOS + NaCl). Each treatment was performed in triplicate. Subsequently, 0.3% NaCl solution was injected periodically and quantitatively every two days. The dosage was three times the water holding capacity to maintain a constant concentration. The control group was injected with the same amount of water. At the two-leaf and one-heart stage, 1/4 Hoagland nutrient solution was irrigated once; 0% NaCl and 0.3% NaCl solutions were prepared with 1/4 Hoagland nutrient solution. IR29 and FL478 seedling leaves were extracted at 24 h after treatment, and the transcriptomic and metabolomic analyses were conducted. Samples were obtained from the plant at the three-leaf and one-heart stage (15 days after AOS spraying). SOD, CAT, APX, MDA, AsA, and GSH levels in the rice leaves were determined. Aboveground and underground dry weights and phenotypes of whole rice were determined.

### Phenotypic indices and biomass analysis

The aboveground portion of rice was used to determine plant height, stem diameter and leaf area. Roots were scanned with a Win RHIZO LA6400XL root scanner and analyzed with Win RHIZO Pro software to determine root length, root surface area, root volume, and root tip number. The rice seedlings were rinsed and incubated at 105℃ for 30 min., dried at 80℃ to constant weight, and the aboveground and root dry weights were measured. The aboveground phenotypes and dry weights of 15 biological replicates were determined for each treatment. Root parameters of 9 biological replicates per treatment were also measured.

### Physiological and biochemical indices analysis

Superoxide dismutase (SOD) levels were measured using the nitro-blue tetrazolium (NBT) method [56]. Catalase (CAT) and ascorbate peroxidase (APX) levels were measured using a spectrophotometer [56]. The malondialdehyde (MDA) content was determined using the thiobarbituric acid method [5], and ascorbic acid (AsA) content was measured using 4.7-diphenyl-1, 10-phenanthroline, (BP) color determination [57]. Glutathione (GSH) levels were analyzed via 5, 5-dithiobis − (2-nitrobenzoicacid)(DTNB) colorimetry [58]. Each sample comprised three independent biological replicates.

### RNA extraction and bioinformatics analysis

Total RNA was extracted from rice seedling leaves via ethanol precipitation using CTAB-PBIOZOL reagent. Qualitative and quantitative analysis of total RNA was performed using a Nano Drop and Agilent 2100 biological analyzer (Thermo Fisher Scientific, MA, USA) [59]. Clean reads were obtained using SOAPnuke (v1.5.2) for raw readings [60]. They were then mapped to the reference genome using HISAT2 (v2.0.4) (Kim, Langmead, & Salzberg, 2015), while Ericscript (Benelli et al., 2012) (v0.5.5) and rMATS (Shen et al., 2014) (V3.2.5) were used to fuse genes and differential splicing genes (DSGS). Clean reads were aligned with gene sets using Bowtie2 (v2.2.5) (Langmead & Salzberg, 2012). P < 0.05 and Log2FC ≥ 1 were used to screen differentially expressed genes using Phyper (https://en.wikipedia.org/wiki/Hypergeometric_distribution) based on hypergeometric analysis using GO for the differential expression of genes (http://www.geneontology.org/) and KEGG enrichment analysis (https://www.kegg.jp/).

Real-time fluorescence quantitative polymerase chain reaction (RT-qPCR) was used to verify the expression of ten randomly selected differentially expressed genes in the transcriptome and the reference gene UBQ5. Each gene carried three independent biological repeats. The detailed list of genes and gene-specific primers is presented in Table S1.

### Metabolite extraction, determination and data analysis

The same materials were used for metabolite analysis and the transcriptome. Six replicates were processed for each sample. Each sample was placed in an Eppendorf tube after grinding at 50 Hz for 5 min with a tissue grinder (JXFSTPRP) and ultrasonicated in a water bath at 4 °C for 30 min, followed by refrigeration at -20 °C for one hour. The samples were then centrifuged for 15 min (14,000 RPM Centrifuge Centrifuge (5430)) using a 0.22 m 𝜇 membrane filter following LC-MS analysis. The metabolites were analyzed using Waters 2D UPLC (waters, USA) tandem Q Exactive high-resolution mass spectrometer (Thermo Fisher Scientific, USA). The Compound Discoverer 3.1 (Thermo Fisher Scientific, USA) software was used for data processing. Metabolomics R software package metaX and metabolome information analysis were used for data preprocessing, statistical analysis, metabolite classification, and functional annotation. Data were preprocessed using PQN (Probability Quotient Normalization), QC-RLSC (Quality Control- robust LOESS signal correction), and CV (Coefficient of Variation). Differential metabolites were screened using the filters VIP ≥ 1, fold change ≥ 1.2 or ≤ 0.83, and p-value < 0.05. The KEGG PATHWAY database was used for functional annotation to identify the major biochemical metabolic and signaling pathways involved.

### Statistical analysis

All data were expressed as mean ± standard error (SEM). IBM SPSS Statistics 26 (SPSS, Inc., Chicago, USA) was used for one-way analysis of variance (ANOVA) analysis, followed by Duncan’s multiple range tests. A p < 0.05 was considered a significant difference. Digital ICONS were compiled in Excel 2016, and Wayne charts were drawn in Origin 2021.

### Electronic supplementary material

Below is the link to the electronic supplementary material.

Supplementary Material 1

## Data Availability

The transcriptomic and metabolomic data presented in this study can be found in online repositories [61, 62]. The raw sequence data have been deposited in the Genome Sequence Archive in National Genomics Data Center, Beijing Institute of Genomics, Chinese Academy of Sciences, under accession number CRA012034 and are publicly available at https://bigd.big.ac.cn/gsa. The metabolite data have been deposited in the National Genomics Data Center, Beijing Institute of Genomics, Chinese Academy of Sciences, under accession number OMIX004644 and are publicly available at: https://bigd.big.ac.cn/omix.
