# Supplementary material for: Combined transcriptomic and metabolomic analysis of alginate oligosaccharides alleviating salt stress in rice seedlings
Source: BMC Plant Biol. 2023 Sep 29;23:455. doi: 10.1186/s12870-023-04470-x (PMC10540332; doi:10.1186/s12870-023-04470-x)
Supplement: Supplementary file 1 — Supplementary Material 1 [file 12870_2023_4470_MOESM1_ESM.docx]

# Supplementary Figures and Tables

Table S1 List of genes and corresponding primers for real-time fluorescence quantitative PCR

| **Gene name** | **Gene ID** | **Primer name and sequence** |
| --- | --- | --- |
| OsPOX1 | Os01g0263300 | Os01g0263300-F2 5' GTTGCCTGTTGATGCTCTGC 3' |
|  |  | Os01g0263300-R2 5' GCCTGTGCTACGATGGACTG 3' |
| OsLECRK2 | Os04g0202300 | Os04g0202300-F1 5' AGGATTGACTTGCTGGTGGC 3' |
|  |  | Os04g0202300-R1 5' TCAGGAGGCGTTGGGATTT 3' |
| OsCER4 | Os04g0354600 | Os04g0354600-F1 5' TCAAGAGATTAGCATACTTCCAGG 3' |
|  |  | Os04g0354600-R1 5' AAGACATACGGGGCAAACAG 3' |
| OsXTH11 | Os06g0696400 | Os06g0696400-F1 5' GGTTCCAGTCCAAGAGCGA 3' |
|  |  | Os06g0696400-R1 5' TGCGACGACAAGTAGAAGGTG 3' |
| CYP86B1 | Os10g0486100 | Os10g0486100-F2 5' TACTACCAGATGAAGTTTGCCG 3' |
|  |  | Os10g0486100-R2 5' TCTTGTCCCTCTTGGTCAGC 3' |
| OsWRKY65 | Os12g0116800 | Os12g0116800-F1 5' GCGAGACTGGTAATTGGAGC 3' |
|  |  | Os12g0116800-R1 5' CCCGCTCATCAAGTCTATCAC 3' |
| OsPRP1 | Os05g0226900 | Os05g0226900-F2 5' CGTGCGATGTACGAGAAGC 3' |
|  |  | Os05g0226900-R2 5' TTAGGTTCTGGATTAGGTTTTGG 3' |
| OsPsbR1 | Os07g0147500 | Os07g0147500-F1 5' GGCTCTGAAACCATCTGCAT 3' |
|  |  | Os07g0147500-R1 5' ACACCTTCCTTGAACACCACTC 3' |
| OsHsp17.3 | Os03g0266900 | Os03g0266900-F2 5' GGAGAAGACGGACAAGTGGC 3' |
|  |  | Os03g0266900-R2 5' GTCGGGCTTCTTGGGCTC 3' |
| OsGlu1 | Os01g0940700 | Os01g0940700-F1 5' TGGCGTTGCTTCCGTTTT 3' |
|  |  | Os01g0940700-R1 5' GGTGAGGGCGATGCTTGA 3' |
| UBQ5 | Os06g0650100 | UBQ5-F1 5' GGAGCGTGTTGAGGAGAAAG 3' |
|  |  | UBQ5-R1 5' CCCTCAGAGCAAGCACGA 3' |

Table S2 RNA-seq profiles of rice leaves after salt stress and AOS treatment

| Sample | Raw Reads（M） | Clean Reads（M） | Clean Bases | Clean Reads Q20 | Clean Reads Q30 | Mapping Ratio | Uniquely Mapping Ratio |
| --- | --- | --- | --- | --- | --- | --- | --- |
| FLCK | 46.74 | 44.64 | 6.70 | 97.73 | 93.51 | 91.04 | 88.79 |
| FLNaCl | 47.91 | 45.67 | 6.85 | 97.50 | 92.96 | 90.67 | 88.41 |
| FLNaCl_AOS | 47.91 | 44.61 | 6.69 | 97.91 | 94.01 | 89.91 | 87.55 |
| IRCK | 45.65 | 43.68 | 6.55 | 97.73 | 93.57 | 90.99 | 88.79 |
| IRNaCl | 46.74 | 44.68 | 6.70 | 97.70 | 93.48 | 91.03 | 88.74 |
| IRNaCl_AOS | 45.57 | 44.04 | 6.61 | 97.66 | 93.31 | 91.17 | 88.87 |


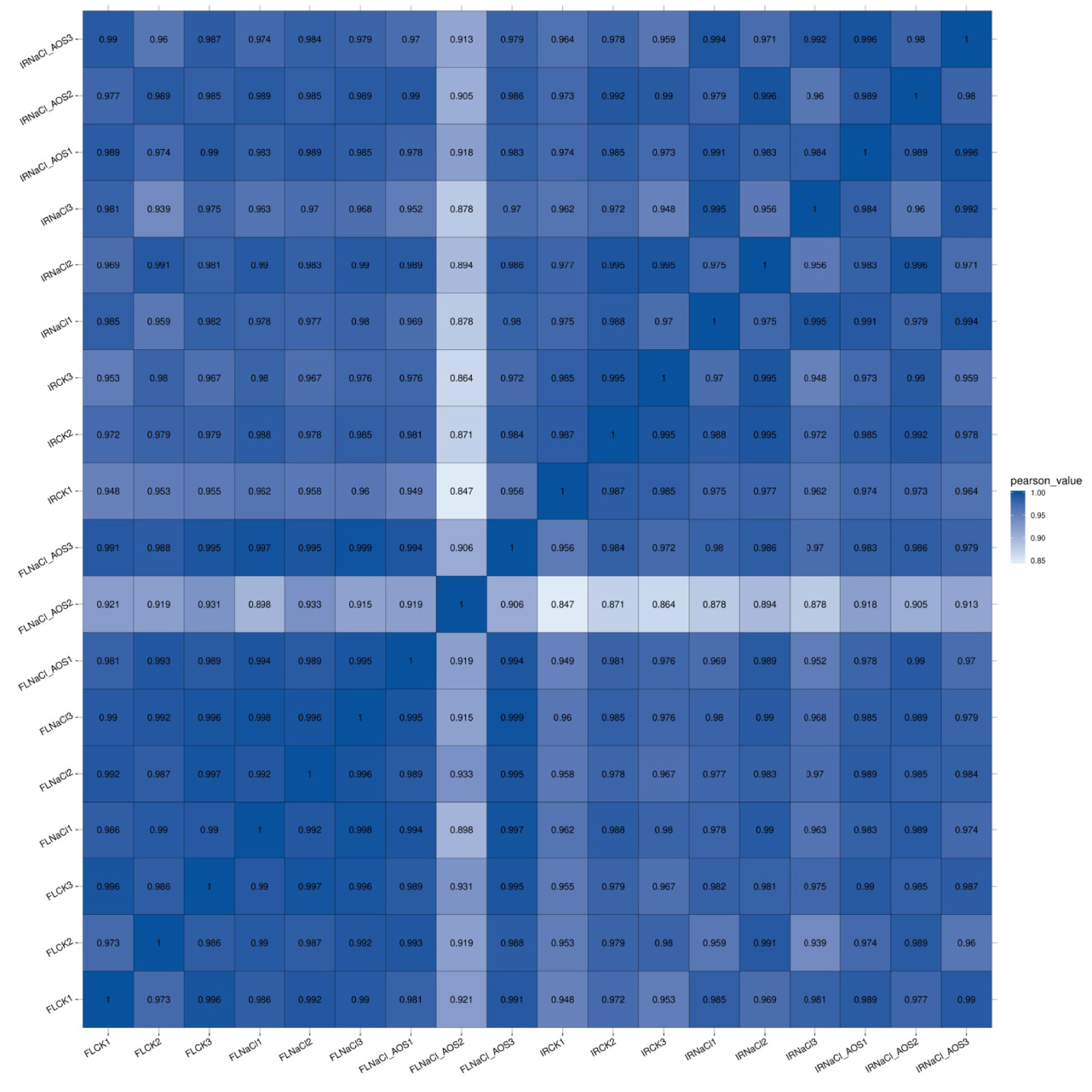


Figure S1 Correlation coefficient between samples

Figure S2. Real-time quantitative PCR (qRT-PCR) verification of gene expression data. The ten differentially expressed genes were detected using qRT-PCR. RNA-ref (A) and qRT-PCR (B) results in FL478 and IR29 rice seedlings. The rice UBQ5 gene was used as an internal standard for qRT-PCR.

Table S3 Up-regulated or down-regulated differential genes in a comparison of alginate oligosaccharides + salts and control rice data.

| Varieties | Gene ID | symbol | log2FC | P-value |
| --- | --- | --- | --- | --- |
| **FL478** | Photosynthesis |  |  |  |
|  | Os03g0439700 | *OsCGLD29* | -1.51 | 5.99155E-06 |
|  | Os09g0346500 | *OsCAB1* | -1.06 | 2.42103E-05 |
|  | Os04g0678700 | *OsPORA* | -1.44 | 0.00265127 |
|  | Os09g0485201 | *OsPSI-M* | 2.02 | 0.009408469 |
|  | Os05g0375600 | *OsPRFB7* | 1.31 | 0.013662994 |
|  | Os06g0176900 | *OsCGS4* | 1.36 | 0.014934419 |
|  | Os09g0502500 | *OsADH9* | 2.42 | 0.017945311 |
|  | Os05g0500450 | *OsADH10* | -1.10 | 0.043004274 |
|  | Os04g0473025 | *Osndhb10* | 1.55 | 0.028809407 |
|  | Os03g0119900 | *OsHisH4* | -1.66 | 0.033415689 |
|  | Os04g0577800 | *OsUOF136* | -1.30 | 0.040135561 |
|  | Os04g0473150 | *Ospsba6* | 1.02 | 0.044584528 |
|  | Os02g0122900 | *OsDIMT2* | 1.03 | 0.048932839 |
|  | Serine/threonine kinase | |  |  |
|  | Os04g0127200 | *OsSub37* | 1.53 | 0.002943506 |
|  | Os02g0153400 | *OsLRK6* | 1.30 | 0.013504496 |
|  | Os02g0215500 | *OsXa21.3* | 1.53 | 0.018917235 |
|  | Os07g0133100 | *OsGALG2* | -3.18 | 0.031426373 |
|  | Os03g0264300 | *OsRLCK106* | 8.04 | 0.039649149 |
|  | Cell wall |  |  |  |
|  | Os02g0588500 | *GDPDL3.1* | 1.20 | 3.78406E-05 |
|  | Os10g0365200 | *OsGSTU33* | -5.72 | 0.000174024 |
|  | Os05g0247100 | *OsXIP2* | -1.32 | 0.000219465 |
|  | Os01g0327100 | *prx15* | -1.83 | 0.016836168 |
|  | Os08g0103900 | *OsSWN3* | 4.02 | 0.025248817 |
|  | Os09g0538000 | *OsRNS5* | -2.73 | 0.026533905 |
|  | Os03g0858600 | *OsDUF668-5* | -3.34 | 0.037434529 |
|  | Os04g0477300 | *NAC81* | 1.17 | 0.044485561 |
|  | Os08g0108100 | *OsPMEI28* | -2.08 | 0.045033022 |
|  | Carbohydrate |  |  |  |
|  | Os01g0950866 | *OsPslp4* | -6.25 | 0.002767483 |
|  | Os02g0105400 | *LDH* | -1.04 | 0.007683567 |
|  | Os02g0528200 | *OsSBEIIb* | 1.19 | 0.019918156 |
|  | Os01g0287600 | *CHT10* | -1.48 | 0.023809295 |
|  | Os12g0131150 | *OsGFAT3* | 3.14 | 0.024655973 |
|  | Os03g0168000 | *OsMFDS14* | -2.93 | 0.048219419 |
|  | Hormone |  |  |  |
|  | Os08g0174800 | *OsBISERK2* | 5.73 | 0.000544728 |
|  | Os01g0764800 | *OsGH3.2* | -3.69 | 0.0027473 |
|  | Os11g0155600 | *OsMRP16* | 1.66 | 0.009578545 |
|  | Os04g0522500 | *OsGA2ox6* | -1.31 | 0.015720587 |
|  | Os03g0810100 | *OsIPT4* | -2.79 | 0.025387623 |
|  | Os07g0136800 | *OsCYP90D4* | 2.51 | 0.031480484 |
| **IR29** | Photosynthesis |  |  |  |
|  | Os03g0592500 | *OsLhcb2.1* | -1.24 | 4.52367E-11 |
|  | Os04g0457000 | *OsLhcb6* | -1.04 | 7.5204E-06 |
|  | Os07g0558400 | *OsLHCB4* | -1.10 | 1.16363E-05 |
|  | Os04g0678700 | *OsPORA* | -1.12 | 1.97964E-08 |
|  | Os01g0600900 | *OsLhcp* | -1.25 | 2.20408E-05 |
|  | Os03g0289400 | *OsStr2* | -1.09 | 4.03411E-05 |
|  | Os04g0414700 | *PSAO* | -1.13 | 8.6014E-05 |
|  | Os09g0294000 | *OshAKHSDH3* | 1.12 | 0.000221142 |
|  | Os06g0694500 | *OsISC13* | -1.09 | 0.000339787 |
|  | Os03g0333400 | *OsPSB27* | -1.26 | 0.000343593 |
|  | Os04g0690800 | *OsPsbS2* | -1.19 | 0.001161188 |
|  | Os08g0423500 | *OsalphaCA3* | 1.04 | 0.000490712 |
|  | Os10g0506200 | *OsValRS3* | 1.16 | 0.00136256 |
|  | Os02g0729400 | *OsStr11* | -1.02 | 0.002236922 |
|  | Os06g0725000 | *OsStr9* | -1.17 | 0.005305255 |
|  | Os06g0132400 | *OsMgMT* | -1.02 | 0.003404068 |
|  | Os02g0122900 | *DIMT2* | 1.11 | 0.008585762 |
|  | Os01g0662300 | *OsRPL12-2* | -1.03 | 0.00883821 |
|  | Os09g0413300 | *OsPPR1* | 1.30 | 0.008914121 |
|  | Os01g0217500 | *OsDJ-1A* | -1.97 | 0.022544359 |
|  | Os06g0199100 | *OsHTH1* | 1.05 | 0.030366317 |
|  | Os04g0432250 | *OsDExH7.1* | 1.43 | 0.048752679 |
|  | Carbohydrate |  |  |  |
|  | Os01g0348900 | *OsJRL* | -2.96 | 2.67131E-10 |
|  | Os05g0316800 | *OsERF056* | -1.10 | 2.59527E-05 |
|  | Os03g0306800 | *OsCP12* | -1.24 | 0.000126244 |
|  | Os01g0713200 | *OsGns10* | -1.02 | 0.001611882 |
|  | Os02g0158500 | *CDC3* | -1.24 | 0.006414848 |
|  | Os02g0577400 | *OsMTEF1* | 1.15 | 0.008644175 |
|  | Os04g0475000 | *Osbglu43* | 1.30 | 0.047726294 |
|  | Serine/threonine kinase | |  |  |
|  | Os03g0146300 | *CKS1* | -1.11 | 4.95061E-05 |
|  | Os04g0420300 | *OsRLCK150* | 1.08 | 0.000144575 |
|  | Os10g0200000 | *OsRLCK295* | 1.22 | 0.010137978 |
|  | Os02g0625300 | *OsPPCK2* | -1.00 | 0.001438697 |
|  | Os05g0457200 | *OsPP2C49* | -1.13 | 0.00174612 |
|  | Os12g0611300 | *OspPLAIIIzeta* | -1.02 | 0.00203877 |
|  | Os11g0173900 | *OsLRR23* | 1.08 | 0.006018899 |
|  | Os02g0234450 | *OsGsSRK2* | 1.17 | 0.012575148 |
|  | Os02g0215700 | *OsXa21.1* | 1.02 | 0.037169717 |
|  | Os02g0216000 | *OsXa21.2* | 4.34 | 0.020407183 |
|  | Os03g0196600 | *OsSAT2;2* | -1.04 | 0.046589011 |
|  | Hormone |  |  |  |
|  | Os02g0703600 | *OsABA8OX* | -1.17 | 1.91819E-11 |
|  | Os09g0457100 | *OsABA8OX3* | -1.01 | 0.000225455 |
|  | Os07g0162700 | *OsCDAP2* | -1.08 | 6.73729E-09 |
|  | Os07g0162900 | *OsCDAP3* | -1.11 | 8.65978E-05 |
|  | Os11g0587000 | *OsD27* | 1.28 | 0.000105009 |
|  | Os02g0769100 | *OsSAUR12* | -1.03 | 0.001903792 |
|  | Os02g0203300 | *OsUGT75J1* | -1.31 | 0.003610496 |
|  | Os01g0699500 | *OsMKKK70* | -1.61 | 0.006692814 |
|  | Os09g0421300 | *OsPLDlambda* | -1.64 | 0.007085684 |
|  | Os12g0443000 | *OsCYP94B5* | -1.82 | 0.015679431 |
|  | Os09g0247700 | *OsBIG* | 1.10 | 0.016210593 |
|  | Os06g0216300 | *OsOPR1* | 2.61 | 0.016529349 |
|  | Os11g0284300 | *PTR* | -1.15 | 0.022273291 |
|  | Os06g0255200 | *OsSYD* | 2.67 | 0.034421927 |
|  | Os07g0421866 | *OsTPR8* | 1.23 | 0.036155839 |
|  | Salt stress response | |  |  |
|  | Os05g0198200 | *OsGRX16* | -1.40 | 8.01088E-06 |
|  | Os11g0655900 | *OsGRX23* | -1.32 | 0.006955341 |
|  | Os01g0135900 | *OsHsp17.9B* | -1.16 | 2.89089E-05 |
|  | Os03g0267200 | *OsHsp17.7* | -1.05 | 0.03982744 |
|  | Os06g0306300 | *OsPRX78* | -1.11 | 3.54366E-05 |
|  | Os02g0192700 | *OsPrxIIE2* | -1.00 | 0.000304924 |
|  | Os08g0113000 | *prx117* | -1.07 | 0.000671213 |
|  | Os07g0694300 | *prx116* | -1.02 | 0.0050964 |
|  | Os10g0109600 | *OsPrx126* | -1.09 | 0.010883123 |
|  | Os02g0833900 | *prx32* | -1.78 | 0.02792461 |
|  | Os09g0507500 | *prx123* | -3.58 | 0.037347461 |
|  | Os03g0368900 | *prx45* | -1.16 | 0.03788233 |
|  | Os09g0544400 | *OsMUC5AC* | 1.82 | 0.003952349 |
|  | Os11g0107400 | *OsMFS6* | 1.15 | 0.008370374 |
|  | Os01g0713900 | *OsMYO17* | 2.09 | 0.015590864 |
|  | Os12g0407200 | *OsTp1* | 1.27 | 0.025331177 |
|  | Os03g0744675 | *OsWD40-90* | 2.12 | 0.027302126 |
|  | Os06g0192800 | *OsATL69* | -1.02 | 0.043593761 |
|  | Transcription factor | |  |  |
|  | Os03g0711100 | *OsCCT14* | -1.34 | 3.14685E-07 |
|  | Os02g0178100 | *OsCCT05* | -1.16 | 0.000378136 |
|  | Os05g0429900 | *OsDLN141* | -1.11 | 3.11059E-05 |
|  | Os03g0624600 | *OsDLN95* | -1.84 | 0.025344958 |
|  | Os04g0493000 | *OsBBX24* | -1.38 | 0.000151011 |
|  | Os04g0423400 | *OsASR4* | -1.23 | 0.000303012 |
|  | Os06g0637500 | *OsMYB102* | -1.03 | 0.000342676 |
|  | Os12g0572000 | *OsMYB91* | -1.16 | 0.046450395 |
|  | Os04g0532800 | *R2R3-MYB* | -1.13 | 0.038090905 |
|  | Os08g0386200 | *OsWRKY69* | -1.10 | 0.00035152 |
|  | Os08g0235800 | *OsWRKY44* | -1.00 | 0.000751461 |
|  | Os01g0946200 | *NAC38* | -1.17 | 0.000375209 |
|  | Os07g0580500 | *OsBZR1* | -1.25 | 0.000453003 |
|  | Os08g0453800 | *Orysa* | 1.55 | 0.007557149 |
|  | Os03g0319000 | *OsVQ10* | -1.07 | 0.007708444 |
|  | Os09g0522200 | *OsERF24* | -1.00 | 0.010165486 |
|  | Os01g0752500 | *OsERF922* | -1.21 | 0.019374054 |
|  | Os01g0566800 | *OsbHLH117* | -1.06 | 0.010392199 |
|  | Os02g0691500 | *OsbHLH049* | -3.32 | 0.020693032 |
|  | Os02g0708500 | *OsSUVR5* | 1.91 | 0.014380032 |
|  | Os06g0719500 | *OsFD6* | -1.74 | 0.015131424 |
|  | Os05g0151000 | *OsDDRP1* | 1.26 | 0.016837744 |
|  | Os09g0409950 | *OsTAF2* | 1.30 | 0.023180785 |
|  | Os05g0500600 | *OsGRAS29* | -1.10 | 0.03820989 |

Table S4 Differential metabolites with significantly up-regulated or down-regulated abundance only in alginate oligosaccharides + salt and control IR29

| Metabolite ID | Metabolite Name | P-value | VIP | Log_2_FC |
| --- | --- | --- | --- | --- |
| 15.242_294.18594 | Myristyl sulfate | 0.00 | 2.39 | -1.02 |
| 11.26_350.20897 | Andrographolide | 0.00 | 1.16 | 0.36 |
| 13.47_266.15464 | Dodecyl sulfate | 0.00 | 2.65 | -1.15 |
| 12.056_353.25663 | 15-epi prostaglandin a1 | 0.00 | 1.23 | -0.30 |
| 11.21_506.12018 | Metaflumizone | 0.00 | 1.26 | -0.30 |
| 5.321_438.17317 | 1-Cyclohexyl-3-[(1S,11aS)-5,11-dioxo-7-(2-thienyl)-2,3,5,10,11,11a-hexahydro-1H-pyrrolo[2,1-c][1,4]benzodiazepin-1-yl]urea | 0.00 | 3.46 | 0.99 |
| 5.548_338.10011 | 4-Methylumbelliferyl-Î±-D-glucopyranoside | 0.00 | 1.69 | 0.44 |
| 11.036_294.18264 | 6-Gingerol | 0.00 | 1.79 | -1.16 |
| 12.056_333.23043 | Stearidonoyl glycine | 0.00 | 1.50 | -0.44 |
| 8.001_308.21985 | PPG n5 | 0.00 | 1.89 | -0.57 |
| 9.908_486.33531 | Quillaic acid | 0.00 | 1.09 | -0.35 |
| 7.485_188.10476 | Azelaic acid | 0.00 | 1.71 | -0.39 |
| 12.903_327.24101 | 10-Nitrooleate | 0.00 | 1.36 | -0.41 |
| 5.256_388.23179 | N-benzyl-1-{(2r,4s,5r)-5-[6-(2-furyl)-2-methyl-4-pyrimidinyl]-1-azabicyclo[2.2.2]oct-2-yl}methanamine | 0.00 | 2.67 | 0.95 |
| 8.973_512.26066 | [(3r,4s)-1-(4-morpholinylcarbonyl)-3-(2-{4-[3-(trifluoromethyl)phenyl]-1-piperazinyl}ethyl)-4-piperidinyl]acetic acid | 0.00 | 1.51 | -0.44 |
| 7.215_324.19366 | 15-(tert-Butyl)-2,3,5,6,8,9,11,12-octahydro-1,4,7,10,13-benzopentaoxacyclopentadecine | 0.00 | 1.07 | -0.31 |
| 9.115_326.04260 | N1-(4-Bromo-3-methylphenyl)azepane-1-carbothioamide | 0.00 | 1.45 | 0.63 |
| 6.933_178.02651 | 5,7-Dihydroxychromone | 0.00 | 1.42 | -0.28 |
| 8.974_528.23515 | Methyl (2R,4S,6S,12bR)-2-[(2-acetamidoethyl)amino]-4-[4-(trifluoromethyl)phenyl]-1,2,3,4,6,7,12,12b-octahydroindolo[2,3-a]quinolizine-6-carboxylate | 0.00 | 1.28 | -0.40 |
| 10.891_191.13128 | DEET | 0.00 | 1.43 | 0.51 |
| 7.674_432.10593 | Vitexin | 0.00 | 1.25 | -0.87 |
| 10.154_371.26722 | Prostaglandin h1 | 0.00 | 1.25 | -0.66 |
| 9.905_314.04186 | Wedelolactone | 0.00 | 1.21 | -0.30 |
| 9.363_307.21470 | 10-Nitrolinoleate | 0.00 | 1.05 | -0.74 |
| 0.771_155.06943 | L-Histidine | 0.01 | 1.59 | 0.35 |
| 16.124_382.27154 | 1a,1b-dihomo prostaglandin f2О± | 0.01 | 1.69 | -0.69 |
| 0.816_542.12407 | Rhusflavanone | 0.01 | 1.32 | -0.41 |
| 8.983_382.21993 | (4as,9ar)-7-(2-acetamidoethyl)-n-allyl-6-oxodecahydro-2h-pyrido[3,4-d]azepine-2-carboxamide | 0.01 | 1.06 | 0.35 |
| 16.473_576.29317 | SR144190 | 0.01 | 2.59 | -1.02 |
| 1.941_478.13131 | (1R,9R)-3-[(E)-2-(4-Chlorophenyl)vinyl]-11-(propylsulfonyl)-7,11-diazatricyclo[7.3.1.02,7]trideca-2,4-dien-6-one | 0.01 | 1.63 | -0.32 |
| 4.772_358.12597 | 3-[2-(ОІ-D-Glucopyranosyloxy)-4-methoxyphenyl]propanoic acid | 0.01 | 2.33 | -0.71 |
| 12.11_334.17518 | Dehydrochloromethyl testosterone | 0.01 | 1.10 | -0.35 |
| 10.073_380.14685 | 5-({[5-(tert-Butyl)-4H-1,2,4-triazol-3-yl]thio}methyl)-3-[3-(1H-pyrrol-1-yl)phenyl]-1,2,4-oxadiazole | 0.02 | 1.33 | 0.61 |
| 4.488_444.22134 | 1-{[(2R,4S,5S)-5-{[Benzyl(methyl)amino]methyl}-1-azabicyclo[2.2.2]oct-2-yl]methyl}-3-(3-thienyl)urea | 0.02 | 1.26 | 0.46 |
| 18.453_767.51710 | Salinomycin | 0.02 | 2.45 | -1.58 |
| 4.837_584.13735 | Neomangiferin | 0.02 | 1.02 | -0.70 |
| 1.151_666.22274 | Stachyose | 0.02 | 1.50 | -0.38 |
| 6.618_400.21119 | N2-{[(3R,4R,5R)-4,5-dihydroxy-3-{[(4-methylphenyl)carbamoyl]amino}-1-cyclohexen-1-yl]carbonyl}-D-leucinamide | 0.02 | 1.06 | 0.30 |
| 14.631_234.16219 | Ageratriol | 0.02 | 1.26 | -0.31 |
| 18.95_426.29582 | N-({(1s,4s,6s)-4-[2-(cyclopentylamino)-2-oxoethyl]-6-isopropyl-3-methyl-2-cyclohexen-1-yl}methyl)-2-methoxybenzamide | 0.02 | 1.08 | -0.28 |
| 0.844_174.01649 | Dehydroascorbic acid | 0.03 | 1.42 | -0.41 |
| 2.833_131.09482 | L-Norleucine | 0.03 | 1.17 | 0.46 |
| 15.46_456.22569 | Vindoline | 0.03 | 1.11 | -0.37 |
| 5.819_510.19354 | Rehmannioside C | 0.03 | 1.15 | 0.57 |
| 4.909_390.19325 | 3-Chloro-4,6-dihydroxy-2-methyl-5-[(2E,6E)-3,7,11-trimethyl-2,6,10-dodecatrien-1-yl]benzaldehyde | 0.03 | 1.85 | 0.68 |
| 2.262_294.12147 | Aspartame | 0.04 | 2.01 | 0.83 |
| 6.025_378.18945 | CUMYL-PICA N-pentanoic acid metabolite | 0.04 | 1.37 | 0.43 |
| 3.737_191.06183 | N-Acetyl-L-methionine | 0.04 | 1.54 | 0.51 |
| 11.778_353.25660 | Prostaglandin F2Î± 1,9-lactone | 0.05 | 3.09 | -1.03 |
| 13.683_334.21200 | 2-Hydroxy-4,5',8a'-trimethyl-1'-oxo-4-vinyloctahydro-1'H-spiro[cyclopentane-1,2'-naphthalene]-5'-carboxylic acid | 0.05 | 1.60 | -0.44 |

Table S5 KEGG annotation pathways of rice metabolites treated with alginate oligosaccharides + salt

| **Varieties** | **Metabolite ID** | **Compounds** | **KEGG annotation** | **KEGG_pathway_annotation** |
| --- | --- | --- | --- | --- |
| FL478 | 2.302_248.11946 | 6-Hydroxymelatonin | C05643 | Tryptophan metabolism（ko00380） |
|  | 1.073_179.07961 | D-(+)-Glucosamine | C00329 | Amino sugar and nucleotide sugar metabolism(ko00520 ) |
|  | 0.844_174.01649 | Dehydroascorbic acid | C05422 | Ascorbate and aldarate metabolism(ko00053) |
|  |  |  |  | Glutathione metabolism(ko00480) |
|  | 9.363_307.21470 | 10-Nitrolinoleate | C13800 | unknown |
|  | 5.726_594.15930 | Vicenin II | C10195 | unknown |
|  | 9.905_314.04186 | Wedelolactone | C10541 | unknown |
|  | 11.85_478.25486 | Diflucortolone pivalate | D03813 | unknown |
|  | 15.159_220.14657 | 2,6-Di-tert-butyl-1,4-benzoquinone | -- | unknown |
|  | 10.011_472.26648 | 4-cyano-n-({(1s,4s,6s)-6-isopropyl-3-methyl-4-[2-(4-methyl-1-piperazinyl)-2-oxoethyl]-2-cyclohexen-1-yl}methyl)benzamide | -- | unknown |
|  | 10.031_490.27684 | N-({(2R,4S,5R)-5-[3-(3,4-Dimethoxyphenyl)-1-methyl-1H-pyrazol-5-yl]-1-azabicyclo[2.2.2]oct-2-yl}methyl)-2-ethylbutanamide | -- | unknown |
|  | 16.124_382.27154 | 1a,1b-dihomo prostaglandin f2О± | -- | unknown |
|  | 17.37_350.16205 | 7-(2,3-Dimethylphenyl)-2-methoxy-2,3-dihydro-1H-pyrrolo[2,1-c][1,4]benzodiazepine-5,11(10H,11aH)-dione | -- | unknown |
|  | 17.766_354.19332 | 2-(3,4-Dimethoxyphenyl)-N-(2-piperidinophenyl)acetamide | -- | unknown |
|  | 7.725_528.18623 | 1,4:3,6-Dianhydro-2-[(benzylsulfonyl)amino]-5-{[4-(4-biphenylyl)-2-pyrimidinyl]amino}-2,5-dideoxy-L-iditol | -- | unknown |
|  | 8.793_376.20933 | 3-[4-(tert-Butyl)anilino]-1-[4-(tert-butyl)phenyl]-2,5-dihydro-1H-pyrrole-2,5-dione | -- | unknown |
|  | 1.642_297.10731 | N(6)-oh-me-adenosine | -- | unknown |
|  | 11.851_330.18063 | (1r,4as)-7-(2-hydroxypropan-2-yl)-1,4a-dimethyl-9-oxo-3,4,10,10a-tetrahydro-2h-phenanthrene-1-carboxylic acid | -- | unknown |
|  | 13.967_334.21199 | (3E)-3-(Hydroxymethyl)-2-oxo-5-[(1S,8aS)-5,5,8a-trimethyl-2-methylenedecahydro-1-naphthalenyl]-3-pentenoic acid | -- | unknown |
|  | 15.16_486.26264 | Andrastin A | -- | unknown |
|  | 7.196_397.14212 | Methyl 2,7,7-trimethyl-5-oxo-4-[3-(2-thienyl)-1H-pyrazol-4-yl]-1,4,5,6,7,8-hexahydro-3-quinolinecarboxylate | -- | unknown |
|  | 7.915_467.22892 | 1-{[(1s,4s,6s)-6-isopropyl-3-methyl-4-{[5-(4-pyridinyl)-1,3,4-oxadiazol-2-yl]methyl}-2-cyclohexen-1-yl]methyl}-3-phenylurea | -- | unknown |
|  | 8.181_467.22889 | N-(3,4-dimethoxybenzyl)-2-[(3r,4s)-3-{[5-(4-fluorophenyl)-1,2-oxazol-3-yl]methyl}-4-piperidinyl]acetamide | -- | unknown |
| IR29 | 0.771_155.06943 | L-Histidine | C00135 | Histidine metabolism(ko00340) |
|  |  |  |  | beta-Alanine metabolism(ko00410) |
|  |  |  |  | Aminoacyl-tRNA biosynthesis(ko00970) |
|  |  |  |  | Biosynthesis of secondary metabolites(ko01110) |
|  | 11.036_294.18264 | 6-Gingerol | C10462 | Stilbenoid, diarylheptanoid and gingerol biosynthesis(ko00945) |
|  |  |  |  | Biosynthesis of secondary metabolites(ko01110) |
|  | 0.844_174.01649 | Dehydroascorbic acid | C05422 | Ascorbate and aldarate metabolism(ko00053) |
|  |  |  |  | Glutathione metabolism(ko00480) |
|  | 7.674_432.10593 | Vitexin | C01460 | Flavonoid biosynthesis(ko00941) |
|  | 11.26_350.20897 | Andrographolide | C20214 | unknown |
|  | 10.891_191.13128 | DEET | C10935 | unknown |
|  | 2.262_294.12147 | Aspartame | C11045 | unknown |
|  | 2.833_131.09482 | L-Norleucine | C01933 | unknown |
|  | 11.21_506.12018 | Metaflumizone | C18523 | unknown |
|  | 3.737_191.06183 | N-Acetyl-L-methionine | C02712 | unknown |
|  | 13.47_266.15464 | Dodecyl sulfate | C08031 | unknown |
|  | 6.933_178.02651 | 5,7-Dihydroxychromone | C09001 | unknown |
|  | 7.485_188.10476 | Azelaic acid | C08261 | unknown |
|  | 15.46_456.22569 | Vindoline | C01626 | unknown |
|  | 18.453_767.51710 | Salinomycin | C15690 | unknown |
|  | 9.363_307.21470 | 10-Nitrolinoleate | C13800 | unknown |
|  | 9.905_314.04186 | Wedelolactone | C10541 | unknown |
|  | 9.908_486.33531 | Quillaic acid | C08972 | unknown |
|  | 10.073_380.14685 | 5-({[5-(tert-Butyl)-4H-1,2,4-triazol-3-yl]thio}methyl)-3-[3-(1H-pyrrol-1-yl)phenyl]-1,2,4-oxadiazole | -- | unknown |
|  | 4.488_444.22134 | 1-{[(2R,4S,5S)-5-{[Benzyl(methyl)amino]methyl}-1-azabicyclo[2.2.2]oct-2-yl]methyl}-3-(3-thienyl)urea | -- | unknown |
|  | 4.909_390.19325 | 3-Chloro-4,6-dihydroxy-2-methyl-5-[(2E,6E)-3,7,11-trimethyl-2,6,10-dodecatrien-1-yl]benzaldehyde | -- | unknown |
|  | 5.256_388.23179 | N-benzyl-1-{(2r,4s,5r)-5-[6-(2-furyl)-2-methyl-4-pyrimidinyl]-1-azabicyclo[2.2.2]oct-2-yl}methanamine | -- | unknown |
|  | 5.321_438.17317 | 1-Cyclohexyl-3-[(1S,11aS)-5,11-dioxo-7-(2-thienyl)-2,3,5,10,11,11a-hexahydro-1H-pyrrolo[2,1-c][1,4]benzodiazepin-1-yl]urea | -- | unknown |
|  | 5.819_510.19354 | Rehmannioside C | -- | unknown |
|  | 6.025_378.18945 | CUMYL-PICA N-pentanoic acid metabolite | -- | unknown |
|  | 8.983_382.21993 | (4as,9ar)-7-(2-acetamidoethyl)-n-allyl-6-oxodecahydro-2h-pyrido[3,4-d]azepine-2-carboxamide | -- | unknown |
|  | 5.548_338.10011 | 4-Methylumbelliferyl-Î±-D-glucopyranoside | -- | unknown |
|  | 6.618_400.21119 | N2-{[(3R,4R,5R)-4,5-dihydroxy-3-{[(4-methylphenyl)carbamoyl]amino}-1-cyclohexen-1-yl]carbonyl}-D-leucinamide | -- | unknown |
|  | 9.115_326.04260 | N1-(4-Bromo-3-methylphenyl)azepane-1-carbothioamide | -- | unknown |
|  | 1.151_666.22274 | Stachyose | -- | unknown |
|  | 1.941_478.13131 | (1R,9R)-3-[(E)-2-(4-Chlorophenyl)vinyl]-11-(propylsulfonyl)-7,11-diazatricyclo[7.3.1.02,7]trideca-2,4-dien-6-one | -- | unknown |
|  | 12.11_334.17518 | Dehydrochloromethyl testosterone | -- | unknown |
|  | 15.242_294.18594 | Myristyl sulfate | -- | unknown |
|  | 16.124_382.27154 | 1a,1b-dihomo prostaglandin f2О± | -- | unknown |
|  | 16.473_576.29317 | SR144190 | -- | unknown |
|  | 4.772_358.12597 | 3-[2-(ОІ-D-Glucopyranosyloxy)-4-methoxyphenyl]propanoic acid | -- | unknown |
|  | 4.837_584.13735 | Neomangiferin | -- | unknown |
|  | 0.816_542.12407 | Rhusflavanone | -- | unknown |
|  | 10.154_371.26722 | Prostaglandin h1 | -- | unknown |
|  | 11.778_353.25660 | Prostaglandin F2Î± 1,9-lactone | -- | unknown |
|  | 12.056_333.23043 | Stearidonoyl glycine | -- | unknown |
|  | 12.056_353.25663 | 15-epi prostaglandin a1 | -- | unknown |
|  | 12.903_327.24101 | 10-Nitrooleate | -- | unknown |
|  | 13.683_334.21200 | 2-Hydroxy-4,5',8a'-trimethyl-1'-oxo-4-vinyloctahydro-1'H-spiro[cyclopentane-1,2'-naphthalene]-5'-carboxylic acid | -- | unknown |
|  | 14.631_234.16219 | Ageratriol | -- | unknown |
|  | 18.95_426.29582 | N-({(1s,4s,6s)-4-[2-(cyclopentylamino)-2-oxoethyl]-6-isopropyl-3-methyl-2-cyclohexen-1-yl}methyl)-2-methoxybenzamide | -- | unknown |
|  | 7.215_324.19366 | 15-(tert-Butyl)-2,3,5,6,8,9,11,12-octahydro-1,4,7,10,13-benzopentaoxacyclopentadecine | -- | unknown |
|  | 8.001_308.21985 | PPG n5 | -- | unknown |
|  | 8.973_512.26066 | [(3r,4s)-1-(4-morpholinylcarbonyl)-3-(2-{4-[3-(trifluoromethyl)phenyl]-1-piperazinyl}ethyl)-4-piperidinyl]acetic acid | -- | unknown |
|  | 8.974_528.23515 | Methyl (2R,4S,6S,12bR)-2-[(2-acetamidoethyl)amino]-4-[4-(trifluoromethyl)phenyl]-1,2,3,4,6,7,12,12b-octahydroindolo[2,3-a]quinolizine-6-carboxylate | -- | unknown |
